# “Alright, that’s enough now, because it’s not fair”: A qualitative study on realities of intersectional inequality among adolescents from Bogotá, Colombia

**DOI:** 10.1186/s12939-026-02902-2

**Published:** 2026-06-04

**Authors:** Johanna Carolina Sánchez-Castro, Nelly Esther Caliz Romero, Laura Pilz González, Katherina Heinrichs, Christiane Stock

**Affiliations:** 1 Institute of Health and Nursing Science, Charité – Universitätsmedizin Berlin, Corporate Member of Freie Universität Berlin and Humboldt-Universität zu Berlin, Augustenburger Platz 1, 13353 Berlin, Germany; 2 Facultad de Enfermería, Universidad Nacional de Colombia, Carrera 45 N° 26-85 Edificio 228, Bogotá, 111321 Colombia

**Keywords:** Intersectionality, Health equity, Social determinants of health, Mental health, Adolescents, Social inequality, Poverty, Social vulnerability, Well-being, Qualitative research

## Abstract

**Background:**

Colombia is one of the most unequal countries in Latin America, with Bogotá exemplifying the urban concentration of structural and intersectional inequalities. Adolescents living in marginalised areas of the city face vulnerabilities due to overlapping systems of oppression, including classism, racism, sexism, and xenophobia. The COVID-19 pandemic further exacerbated these disparities, heightening socioeconomic stressors and influencing developmental processes. This study aimed to explore how adolescents in two socioeconomically disadvantaged neighbourhoods in Bogotá experience and engage with intersectional inequality in their everyday lives, particularly in the context of the pandemic.

**Methods:**

The study employed a qualitative design. Data were collected through problem-centred semi-structured interviews with adolescents and school counsellors, complemented by direct observation. Participants were recruited from two schools located in areas characterised by marginalisation and social exclusion. Interviews explored perceptions of social inequality, mental health, and the influence of COVID-19 measures. Data were analysed using thematic analysis, following a collaborative, iterative process involving multiple researchers to ensure reflexivity. National and international ethical guidelines were followed in the development of the research.

**Results:**

The synthesis of 95 h of observation notes, 42 participating adolescents (aged 12–18, 57% girls), and six professionals (mainly psychologists) revealed three overarching themes: (a) perceiving inequality, wherein adolescents experience multiple, intersecting forms of social inequality, intensified by the pandemic; (b) feeling inequality, which highlights how the structural disadvantages were linked to emotional distress, reduced future expectations, and strained family dynamics; (c) becoming a social agent of change. The data showed that despite these adversities, many participants demonstrated agency by using peer support networks and creative expression to cope with social injustice.

**Conclusions:**

The findings highlight the importance of addressing adolescent life experiences, feelings and behavioural performance through an intersectional lens, particularly in urban contexts marked by structural disadvantage. Interventions must consider the interconnected nature of social inequalities and prioritise the voices of young people in policy and programme development. Future research should deepen the understanding of adolescent experiences of inequality and promote inclusive strategies for psychosocial support.

**Supplementary Information:**

The online version contains supplementary material available at 10.1186/s12939-026-02902-2.

## Non-English abstract (Spanish)

**Antecedentes:**

Colombia es uno de los países más desiguales de América Latina, y Bogotá ejemplifica la concentración urbana de desigualdades estructurales e interseccionales. Los adolescentes que viven en barrios marginados enfrentan múltiples vulnerabilidades derivadas de sistemas de opresión como el clasismo, racismo, sexismo y xenofobia. La pandemia por COVID-19 profundizó estas disparidades, intensificando el estrés socioeconómico y afectando procesos de desarrollo. Este estudio exploró cómo los adolescentes en dos barrios de Bogotá experimentan y responden a la desigualdad interseccional, particularmente en el contexto de la pandemia.

**Método:**

Se utilizó un enfoque cualitativo. Los datos se recolectaron mediante entrevistas semiestructuradas centradas en el problema con adolescentes y orientadores escolares, complementadas con observación directa. Los participantes fueron reclutados en dos colegios ubicados en zonas de alta vulnerabilidad social. Las entrevistas abordaron percepciones sobre desigualdad social, salud mental y el impacto de las medidas asociadas al COVID-19. El análisis temático reflexivo se desarrolló de manera iterativa y colaborativa, siguiendo principios éticos nacionales e internacionales.

**Resultados:**

A partir de 95 horas de observación, 42 entrevistas a adolescentes (12 a 18 años, 57% mujeres) y seis entrevistas a profesionales (principalmente psicólogas), se identificaron tres temas principales: (a) percibir la desigualdad, en donde los y las adolescentes reconocen múltiples formas de desigualdad social, agravadas por la pandemia; (b) sentir la desigualdad, que evidencia la relación entre estas condiciones y el malestar emocional; y (c) convertirse en agentes sociales de cambio, mostrando cómo, a pesar de las adversidades, muchos participantes ejercen agencia mediante redes de apoyo entre pares y expresión creativa para enfrentar la injusticia social.

**Conclusiones:**

El estudio destaca la necesidad de abordar las experiencias adolescentes desde una perspectiva interseccional, especialmente en contextos urbanos marcados por desigualdad estructural. Las intervenciones deben considerar la interconexión entre desigualdades y dar protagonismo a las voces juveniles en la formulación de políticas y programas. Se recomienda profundizar en el estudio de estas experiencias y promover estrategias inclusivas de acompañamiento psicosocial.

## Introduction

Social inequality is a structural condition generated and sustained by the capitalist socio-economic model, which is centred on the accumulation of power and wealth [1]. According to the Social Determination of Health framework [1], this model produces an uneven distribution of resources, opportunities, and living conditions across different social groups. As a result, these conditions produce social injustice and deep disparities between those who hold various forms of power (economic, political, or social) and those who lack the means to secure fundamental rights such as access to healthcare, education, adequate housing, and decent employment [1, 2]. To fully understand the complexity of social inequality, it is essential to adopt an intersectional perspective. As proposed by Black feminist movements, this approach recognises how systems of privilege and oppression, such as classism, sexism, heterosexism, and racism, intersect to shape individuals’ lived experiences and access to opportunities [3, 4]. Individuals simultaneously occupy multiple socially constructed identities, based on class, gender, sexual orientation, disability status, ethnicity, among others, which are not isolated but interdependent and mutually constitutive [5]. These intersecting identities produce unique social experiences that cannot be adequately explained by examining axes of inequality in isolation. Therefore, this study adopts an intersectional approach that moves beyond narrow definitions of disadvantage, whether economic, social, or cultural, and instead focuses on how multiple systems of oppression operate in an interwoven manner to produce and reproduce structural injustice [6].

Applying this intersectional lens to the Colombian context is particularly relevant given the country’s profound and persistent inequalities. According to the Economic Commission for Latin America and the Caribbean (ECLAC) [7], Colombia is one of the most unequal countries in the world and ranks as the second most unequal nation in the Latin American and Caribbean region, following Brazil. These disparities are not isolated but are reproduced through the interlocking of socioeconomic structures and longstanding systemic conflicts [8]. In 2019, 17.5% of the population were considered multidimensionally poor, facing deprivation in several basic aspects of a dignified life, such as education, healthcare, housing, and access to essential services [9, 10].

Socioeconomic inequality intersects with persistent gender inequalities, creating a phenomenon of feminisation of poverty where women are disproportionately represented among the most marginalised populations [11, 12]. Despite the existence of legal frameworks designed to protect women’s rights [13, 14], patriarchal norms, *machismo*, misogyny, and homophobia continue to prevail. These structural belief systems reinforce the idea that what is masculine and heteronormative is inherently superior, more capable, and more valuable than that which is feminine or non-normative [15, 16]. This is not merely reflected in wage disparities, but in a systemic interlocking of gender and class that restricts women’s economic autonomy [14, 17]. Furthermore, alarmingly high rates of gender-based violence exacerbate the social injustice experienced by women, girls, and lesbian, gay, bisexual, transgender, intersex, and queer (LGBTIQ+) populations, deepening their marginalisation [16, 18].

Social hierarchies based on racialization further compound this landscape of intersecting inequality. In a world where whiteness and “*mestizaje*” (a Latin American construct referring to the idealised mixing of European and Indigenous ancestry) are socially constructed as ideals of humanity, individuals with darker skin tones, particularly those of Afro-descendant origin, are often relegated to the lowest rungs of social stratification [19]. These hierarchies do not operate in isolation; they synergistically produce discriminatory practices of segregation and exclusion rooted in phenotypic traits [20]. Similarly, xenophobia operates as another axis of oppression, targeting individuals based on their country or region of origin, such as the numerous people who migrated from Venezuela [21]. In Colombia, these interlocking systems of power do not merely add disadvantages; they create a unique landscape of vulnerability where marginalised populations are exposed to systemic invisibilization and the denial of their fundamental rights [21–23].

The structural and intersecting inequalities already present in Colombian society were further exacerbated by the COVID-19 pandemic, which saw monetary poverty rise sharply from 35.7% to 42.5% [24]. Beyond these economic indicators, the crisis deepened non-monetary dimensions of inequality through prolonged lockdowns that intensified domestic violence, job insecurity, and the deterioration of household wellbeing [25–27]. In Bogotá, the nation’s political and economic epicentre, these disparities are deeply embedded in the urban fabric through socio-spatial segregation [28, 29]. This spatialization represents a material intersection of class, labour informality, and restricted access to services that disproportionately affects marginalised areas [16, 20, 30, 31]. Consequently, the pandemic did not merely add new hardships but actively reconfigured the interlocking vulnerabilities of historically marginalised groups, reinforcing the spatial and structural nature of intersectional inequality across the city.

Growing up in such a fragmented urban environment entails challenges where inequality is inscribed into daily life. For adolescents, this critical developmental stage is not experienced uniformly; rather, it is mutually constituted by the structural constraints of their surroundings [32]. In Bogotá, navigating adolescence within structurally unequal contexts does not simply heighten vulnerability; it creates qualitatively different life trajectories depending on how a young person’s gender, socioeconomic status, and place of residence intersect [2, 32]. From a collective mental health perspective, these psychosocial burdens are not viewed as individual clinical disorders, but as forms of social suffering and emotional distress that are intrinsically tied to the city’s systemic patterns of exclusion and marginalisation [33, 34].

Given the complexity of structural and social disadvantage in urban contexts such as Bogotá, it is essential to analyse social inequality through an intersectional lens that captures the interlocking of multiple systems of oppression. Adolescence, as a formative and highly sensitive stage of life, is especially shaped by these dynamics. Investigating how young people experience, interpret, and respond to such inequalities is critical for understanding their life experiences and developmental pathways. Moreover, the COVID-19 pandemic substantially exacerbated existing structural inequalities, disproportionately affecting marginalised populations and intensifying the precarious conditions faced by many adolescents and their families. Against this backdrop, the present study aims to explore how adolescents living in Bogotá, one of Latin America’s most unequal urban settings, experience and engage with intersectional forms of inequality, particularly in the context of the COVID-19 pandemic.

## Methods

### Study design and setting

The study was informed by a social constructionist perspective, which holds that knowledge and meaning are co-constructed through social interaction and shaped by historical and cultural contexts [35]. From this standpoint, adolescents’ accounts were treated as situated understandings shaped by their positionalities within Bogotá’s socio-spatial hierarchy. The research was initially grounded in the Social Determination of Health framework [1], which provided a structural lens to address the multidimensional production of inequality. However, as data collection and analysis progressed, the limitations of a solely structural reading became apparent. In response, an intersectional lens was integrated inductively into the analytic process [3, 4, 36], allowing us to examine how processes of social determination are lived and reconfigured across the life course through interlocking systems of power that shape adolescents’ everyday realities within their territories.

To operationalise this framework, we adopted a qualitative design and reflexive thematic analysis [37], focusing on how intersecting structures of social determination shape adolescents’ narratives. Fieldwork was conducted in three public schools that agreed to participate in the study, located in Bosa and Tunjuelito, two socioeconomically disadvantaged districts in Bogotá, Colombia. These neighbourhoods report multidimensional poverty rates of 11.4% and 9.6%, respectively, significantly higher than the citywide average of 4.3% [28, 38].

Data collection was conducted in successive and partially overlapping phases. Fieldwork, including both observation and interviews, was carried out on weekdays between October 2022 and June 2023, excluding the end-of-year school holiday period (approximately the last two weeks of December and the first two weeks of January). The fieldwork began with four weeks of direct observation within the schools, which allowed the researcher to become familiar with institutional routines, spatial arrangements, daily interactions, and the broader social context of potential participants [39, 40]. This initial phase informed the subsequent recruitment process and the conduct of problem-centred interviews, which followed a semi-structured interview guide designed to elicit adolescents’ personal experiences and perspectives in their own words [41–43]. During the interview phase, observation continued intermittently when participants were unavailable. Following completion of the interviews, additional observation were conducted in the surrounding neighbourhoods to further contextualise adolescents’ accounts.

Analytically, interviews provided in-depth insight into adolescents’ lived experiences, while observation contributed contextual understanding and enabled comparison between participants’ accounts and everyday practices. Interviews with adolescents and school counsellors and observational data were analytically integrated as complementary perspectives to generate a richer and more nuanced understanding of adolescents’ experiences across institutional and neighbourhood contexts. During analysis, interview transcripts and fieldnotes were treated as part of a single qualitative corpus and coded together, allowing observational insights to inform the interpretation and development of themes alongside participants’ narratives. In line with a reflexive thematic analysis approach, themes were developed across the dataset as a whole rather than being attributed to specific data sources, with both interviews and observations contributing to a shared interpretative process.

As a guide for reporting the results of this study, we used the Standards for Reporting Qualitative Research (SRQR) [44].

### Participants and recruitment

Guides were developed for both the documentation of field notes from direct observation and for conducting the interviews. JCSC (principal researcher) prepared both sets of tools and subsequently discussed and refined in collaboration with the research team (NECR, LPG, KH, and CS) (see Additional files 1, 2 and 3). JCSC completely conducted data collection in the participants’ native language, Spanish, which began in October 2022 and concluded in June 2023. This study received ethical approval from Charité – Universitätsmedizin Berlin (application number EA2/056/22, date of approval 09.05.2022) and the Secretaría de Salud Distrital - Bogotá, D.C. (project code SDSCTI20220011, date of approval 19.10.2022).

Adolescents were recruited through an in-school distribution of leaflets containing general information about the study. Interested persons were provided with an informed consent form, which also required their parents’ signature. As for the counsellors, they were approached in their offices, informed about the study, and asked whether they were interested in participating. Those who agreed were required to sign an informed consent form. The inclusion criteria for participants were as follows: adolescents aged 12 to 18 years, of any gender, attending the public schools in Bogotá participating in the study, or counsellors of any gender and age who work in the same schools and were involved in promoting (mental) health among adolescents in these institutions. There were no exclusion criteria.

### Direct observation

A non-participant direct observation technique was employed to explore adolescents’ daily experiences within the school setting, without intentionally influencing their behaviour [39, 40]. Accordingly, JCSC moved through school by walking through the school corridors, entering classrooms, attending parent–teacher or student(s)-teacher(s) meetings when permitted by teachers and coordinators, as well as sitting near groups of adolescents, at times engaging in casual conversations with teachers, coordinators, or the adolescents themselves. Adolescents were not explicitly informed about the observation process; however, when asked, the researcher clarified her role as an observer. To contextualise this observation, JCSC also visited surrounding neighbourhoods (streets, parks, shops) to better understand the adolescents’ living environments. For safety, these visits were conducted with accompaniment and motorised transport. Observations were documented in a field diary, including descriptions of relevant interactions (such as discourses and conversations), as well as impressions, preliminary reflections, and theoretical notes related to the research question.

### Interviews

Before beginning each interview, demographic information was collected from participants using a short standardised questionnaire. The questions were asked verbally, and the responses were registered by the interviewer on a paper form. For adolescents, this included age, gender, place of origin, family structure, primary caregiver, family educational background, housing conditions and access to digital connectivity (e.g., Wi-Fi at home). For counsellors, data were gathered on gender and professional experience. As an icebreaker, participating adolescents were first asked to describe their daily routine. Afterwards, the interview addressed the following topics, each introduced with a guiding question and followed by potential follow-up questions: (a) familiarity with social inequality; (b) emotional responses related to social inequality; (c) awareness of COVID-19 control measures; (d) perceived impact of both COVID-19 measures and social inequality on adolescents’ lives; (e) general aspects of mental health; and (f) changes in adolescent mental health concerning social inequality and pandemic-related restrictions [41–43]. All questions were posed in an open-ended format to encourage participants to express their thoughts and experiences freely [45]. Interviews with participating counsellors began with a general question about their professional role. The same core topics were then explored, with counsellors asked to reflect on these issues concerning adolescents, based on their professional experience. Always, at the end of each interview, participants were invited to share any additional thoughts or relevant information.

The interviews were conducted face-to-face in quiet, unoccupied rooms provided by the institution, such as vacant classrooms, meeting rooms, or counsellors’ offices, ensuring comfort, privacy, and confidentiality during the conversations, following ethical research standards. The sample size was determined based on the concept of information power [46]. Information power was assessed iteratively during data collection, considering the specificity of the study population, the intersectional analytical framework, and the depth and relevance of the narratives obtained. Although the study aim was broad, data collection was concluded when interviews consistently yielded rich, analytically meaningful accounts that adequately addressed the research questions. All interviews were audio-recorded using the same device, which was not connected to the internet.

### Data analysis

We conducted a reflexive thematic analysis at a latent level, aiming to explore the underlying assumptions, ideologies, and power structures embedded in adolescents’ accounts [37]. The coding and theme development process was inductive (data-driven), allowing patterns to be developed directly from the narratives while being critically interpreted through the lens of intersectionality and social determination. Consequently, the analysis was not constrained by a rigid coding frame, but rather evolved to capture the complexity of the participants’ experiences [37, 47, 48].

The analytic process involved several iterative and overlapping phases. First, all fieldnotes were compiled and organised chronologically and prepared for analysis alongside the interview transcripts. Interviews were assigned unique identifiers (A+number for adolescents and C+number for counsellors). Half of the interviews were transcribed manually in Spanish by an independent professional transcriber, and the remaining interviews were transcribed manually by JCSC. All transcripts were subsequently reviewed by JCSC while listening to the audio recordings to correct transcription errors and to remove identifying information.

Second, two researchers (JCSC and NECR) independently engaged with approximately one quarter of the dataset, including both interviews and fieldnotes. This subset was purposively selected to reflect variation in participants’ gender, age, and the depth and diversity of their narratives, following an initial familiarisation with the full dataset. This process supported the initial development of a provisional coding framework and enabled critical discussion of coding decisions, areas of convergence and divergence, and emerging analytical directions. These discussions informed an iterative process of code refinement and early theme construction.

Third, drawing on this provisional framework while remaining open to further inductive development, JCSC conducted the interpretative coding of the remaining data. Throughout this phase, analytic decisions, reflections, and uncertainties were documented in analytic memos to support reflexivity.

In the subsequent phases, JCSC actively constructed candidate themes by clustering conceptually related codes across the dataset in relation to the research question. This involved repeated review and reorganisation of coded extracts to ensure coherence within and distinction between developing themes. Visual mapping techniques were used to explore relationships among themes; these were initially developed manually and later refined digitally using Miro (miro.com). The thematic structure was then reviewed through re-engagement with the full dataset to assess its adequacy and analytical coherence.

Regular analytic meetings were held between JCSC and members of the wider research team (NECR, LPG, KH, and CS) throughout the analysis. These meetings provided a structured space to explain and interrogate emerging interpretations, review draft themes and analytic write-ups, surface and critically examine assumptions, and explore alternative ways of making sense of the data. This ongoing collaborative engagement supported methodological rigour through intersubjective reflexivity, understood as critical dialogue rather than a procedure for establishing consensus or validation [37]. As such, the analysis is understood as a situated and interpretive account shaped by the first author’s analytic engagement with the data, rather than a product of consensus-based procedures [37].

The final analytic narrative was developed by JCSC and subsequently reviewed and refined in collaboration with the broader team. This narrative integrated illustrative data extracts to illuminate each theme and subtheme [37, 47, 48].

Data analysis was primarily conducted in Spanish, the language of data collection. The iterative development of codes and themes occurred in both Spanish and English, reflecting the linguistic composition of the research team. Where necessary for team discussions and for reporting, selected data extracts were translated into English. JCSC decided to use ChatGPT (OpenAI, 2025) as a preliminary tool to support the translation of pseudonymised interview excerpts from Spanish into English. All translations were first carefully reviewed and checked by JCSC to ensure semantic, conceptual, and contextual accuracy before being independently revised and refined by LPG, a native Spanish speaker fluent in English. This multi-step process ensured that the translated quotations faithfully reflected participants’ meanings while minimising potential distortions associated with automated translation tools.

MAXQDA 24.0.0 (VERBI Software, Berlin, Germany) [49] was used to facilitate data management, the coding process, and the iterative grouping of codes into overarching themes.

The principal researcher (JCSC) is a Colombian heterosexual woman from Bogotá, trained as a nurse at a public university in the city and residing in Berlin, Germany during the study. Her dual positionality as both insider and outsider enabled a reflexive analytic stance, grounded in familiarity with local realities and enriched by critical distance. The wider research team brought interdisciplinary perspectives from nursing, psychology, and public and global health, contributing to analytical depth and theoretical coherence [37, 47, 48].

## Results

The data collection process began with an initial phase of direct observation, lasting 79 h in 21 sessions, distributed across the three schools. Subsequently, 16 h in two sessions of observation were carried out in the neighbourhoods mentioned by participants, mostly within the same or nearby districts. Additionally, a total of 42 semi-structured interviews were conducted. Participants’ characteristics are shown in Table 1. The participating adolescents were aged 12 to 18, the majority of whom (57%) were female, heterosexual, and born in Colombia. Most lived in single-parent households, typically under the care of their mothers. Most caregivers had completed only secondary education. Participants generally lived in rented flats with internet access. In parallel, six school counsellors, mostly female psychologists from the same institutions were also interviewed.

**Table 1:** Participants’ demographics (*n* = 48)

Characteristic	Details	Number
Adolescents (*n* = 42)		
Age in years	12	5
	13	7
	14	7
	15	7
	16	10
	17	3
	18	3
Gender	Female	24
	Male	18
Sexual orientation	Heterosexual	33
	Do not know yet	5
	Bisexual	3
	Lesbian	1
Country of birth	Colombia	39
	Venezuela	3
Type of family*	Single parent	13
	Extensive	10
	Nuclear	10
	Composite	9
Main caregiver(s)	Mother	25
	Mother and father	11
	Father	5
	Grandmother and father	1
Highest schooling of primary caregivers	5th grade	3
	7th grade	1
	8th grade	2
	9th grade	8
	High school	18
	Technician training	7
	Bachelor	3
Housing ownership	Familiar	3
	Own	10
	Rent	29
Housing cohabitation**	Single-family housing	40
	Shared housing	1
	Shared sanitation facilities	1
Internet access (Wi-Fi)	No	11
	Yes	31
Counsellors (*n* = 6)		
Gender	Female	5
	Male	1
Profession	Psychologist	5
	Special educator	1

*Single parent: one caregiver; Extended: includes members of different generations (e.g., grandparents, aunts/uncles); Nuclear: two parents and their children; Composite: includes multiple nuclear units or stepfamilies (e.g., mother, stepfather, and children)

**Fig. 1:**
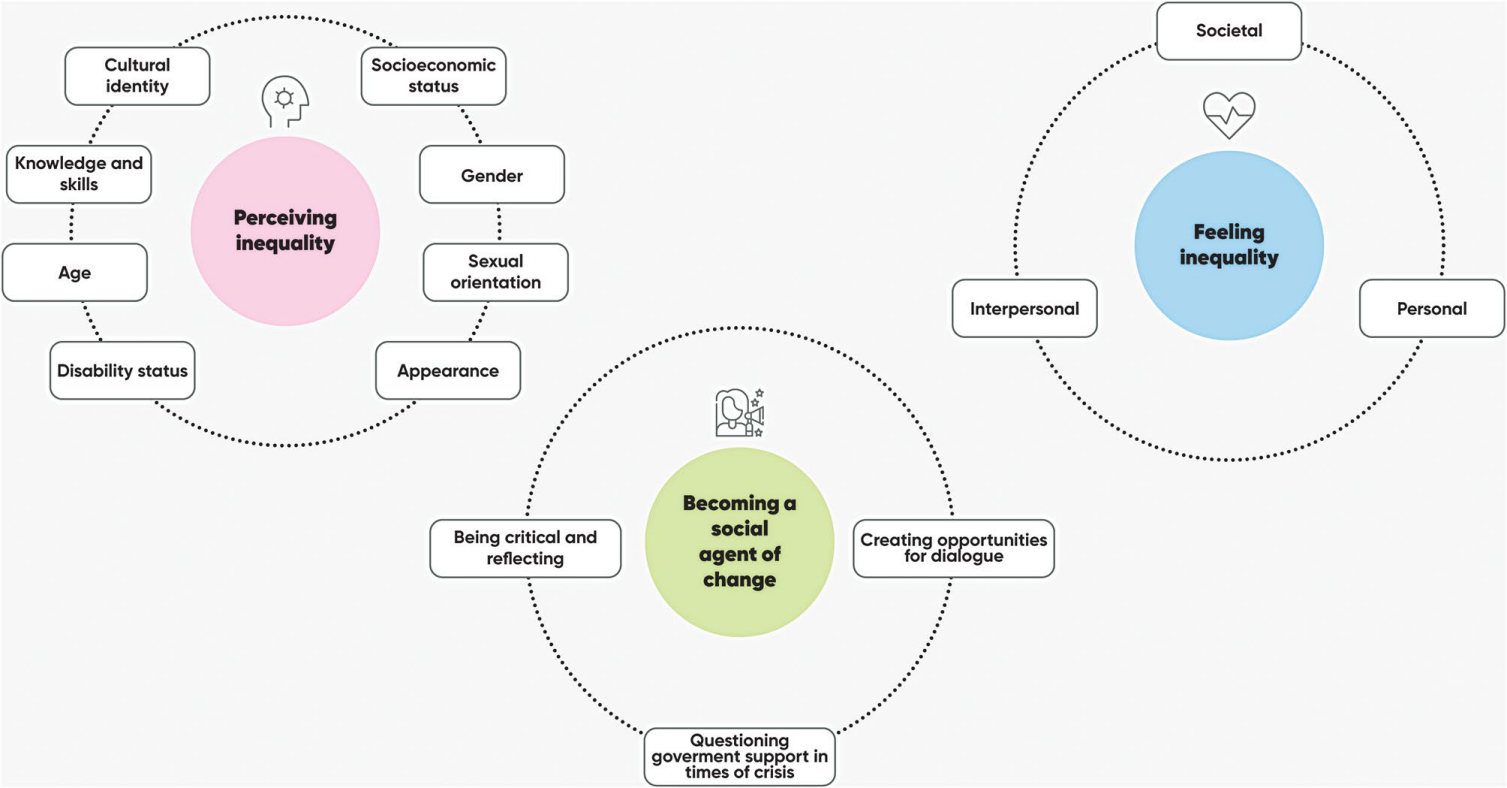
Adolescents’ experience of intersectional inequality with three overarching themes and sub-themes for each theme. Source: author’s own elaboration (designed on miro.com)

The results of the thematic analysis illustrate how adolescents experience intersectional inequality across the various contexts they navigate. This is explained through three overarching themes: (a) perceiving inequality, (b) feeling inequality, and (c) becoming a social agent of change, each containing subthemes that provide a deeper understanding (see Fig. 1). All quotes used to illustrate the results are also presented in the original language (Spanish) in the Additional file 4.

### Perceiving inequality

This section outlined the different forms of inequality reported by participants, highlighting the specific mechanisms through which they were marginalised. Following an intersectional logic, these subthemes were not interpreted as discrete or independent variables; rather, they represented multiple axes of a single socio-spatial hierarchy in Bogotá. Each subtheme provided a specific entry point through which adolescents perceived the interlocking nature of structural violence in their daily environments.

#### Socioeconomic status

The narratives revealed that for adolescents in Bosa and Tunjuelito, wealth distribution was not merely a background variable but a force that reconfigured their daily existence. Scarce resources, exacerbated by the COVID-19 pandemic, impacted their physical and mental health, restricted social opportunities, and reinforced structural marginalisation. Most participants lived in households headed by single mothers engaged in precarious, informal employment, where income often failed to cover essential needs.

*C1 (female*,* psychologist): A student described it to me like this:*,* “Teacher*,* I realised I was poor when the pandemic hit*,* because before that*,* I hadn’t realised I was poor*,* because I went to school and there I was distracted with my classmates*,* they [the school] gave me food there*,* I got uniforms from donations and stuff. But being stuck at home*,* there was no food. My mom lost her job*,* my dad lost his job*,* everyone was just there*,* at home” […] so*,* from there*,* you can really see how the system of inequality and unfairness works.*

Food insecurity was a recurring theme, linked to both low household income and rising prices. While school meals provided a vital buffer, the lockdown exposed the fragility of this support. Participants described extreme survival strategies, such as borrowing money for basic sustenance or, in one critical account, consuming newspaper to dull hunger. Housing further reflected this hardship; many resided in subdivided commercial spaces or precarious dwellings where high rents consumed a substantial share of income, leading to overcrowding or forced relocations. Relationships between tenants and landlords can be strained, with the latter sometimes imposing behavioural restrictions on adolescent residents.

Living in the city’s periphery imposed a ‘poverty tax’ of time and money. Adolescents described waking up as early as 4:00 a.m. to navigate long, costly, or unsafe commutes, often balancing these with domestic care burdens (e.g., preparing meals for working caregivers). This spatial segregation directly impacted their well-being, as public transport was perceived as unreliable and difficult to access.

*A12 (female*,* 16 years old): Right now*,* we’re not doing too well. My mum*,* well*,* she got a slight raise*,* but it’s still not enough because we have to pay for*,* let’s say*,* the flat*,* […] the service charge. We also have to pay for food and utilities […] food is very expensive*,* too expensive. I mean*,* you see something and think*,* “Oh*,* that’s quite cheap*,*” but when you add everything up*,* it’s extremely expensive. My mum… right now*,* we don’t have a great financial situation to buy loads of food*,* like a really big purchase […]. We don’t have much to eat*,* but we’re not really struggling or starving*,* because my brother’s godparents live here too [godparents are the social and economic support network for the adolescent’s family].*

Participants also experienced limited institutional responsiveness, particularly within healthcare systems, where long waiting times and brief consultations were common. One participant mentioned, for example, receiving sleeping pills while only being able to schedule an appointment in three months. Private healthcare was seen as a more effective alternative, but one financially inaccessible to most families. Regarding educational trajectories, higher levels of schooling were often perceived as unattainable unless basic needs were met. Participants acknowledged the structural advantages of affluent peers, perceiving education both as a route to social mobility and a source of stress due to potential debt. Furthermore, the digital divide during the pandemic turned education into a privilege, effectively excluding those without internet or devices from the right to learn.

*A8 (female*,* 16 years old): When it comes to getting into university*,* a person with resources can easily enrol in a professional degree programme*,* like medicine*,* and start their studies straight away. Meanwhile*,* I have to look for scholarships*,* figure out which university I can afford*,* so I don’t end up in debt*,* for example*,* with a loan*,* because that could really stress me out. So*,* it’s that*,* it directly affects me*,* because not having the right socio-economic background means I can’t fully meet my needs.*

#### Gender

Female adolescents expressed dissatisfaction with the unequal distribution of household tasks, a situation that became even more pronounced during the COVID-19 lockdown. Girls were expected to take on a disproportionate share of domestic responsibilities such as cooking, cleaning, and caregiving, despite the presence of equally capable male family members (e.g., brothers, fathers, uncles, or cousins), who were often exempt from such duties. Crucially, this domestic confinement was intertwined with socioeconomic necessity, as girls’ free labour often sustained the household while mothers worked in informal sectors. This unequal burden limited girls’ time for personal interests. Moreover, they were subject to stricter social controls, including restrictive curfews and prohibitions on social outings. Caregivers frequently pressured them to form heteronormative relationships considered ‘appropriate’ and expected them to support their mothers in daily life, even when it conflicted with their own needs or desires, expectations rarely imposed on male relatives.

*A42 (female*,* 18 years old): Lately*,* it’s always like*,* “Oh*,* poor him*,* he needs this*,*” or “poor thing*,* he has to do that because he works.” Like*,* “Oh*,* we need to let him rest”*,* or “we need to have his food ready.” For example*,* my brother never does any housework*,* he never even picks up a broom*,* but my granny and my mum do because they are women. I have to pick up a broom and do the chores*,* and I can’t do what they [male family members] do*,* just go out and leave everything messy*,* or leave a plate of food lying around. You just can’t do that. And they’ve even said it to my face many times: “But he’s a man*,* and you’re a woman*,* and women do this.” So …*.

Participants reported instances in which girls were criticised for expressing physical strength, while boys were discouraged from displaying emotional vulnerability, such as sensitivity or insecurity. These gender norms were reinforced at home, where some adolescents had been told that groups of women in the workplace were untrustworthy due to gossip. One participant shared that her father abandoned her because he had hoped for a boy.

*A12 (female*,* 16 years old): That had a big influence on my life here at school*,* because they [classmates] used to call me “marimacha” [Spanish for tomboy]*,* like*,* “the boys will see you*,*” and yeah*,* it was true*,* but still*,* sometimes they would exclude me in such a horrible way*,* and I hate being excluded*,* or seeing someone else being excluded*,* because I know exactly how that feels.*

Furthermore, adolescents also recognised how their mothers were subjected to male dominance through gender-based violence. Many had witnessed fathers or stepfathers perpetrating verbal, physical, psychological, and economic abuse against their partners. Their accounts included episodes of domestic conflict involving physical and verbal aggression, as well as instances where men deliberately restricted women’s access to financial resources, thereby fostering economic dependence or deliberately destroying their partner’s property, illustrating how patriarchy and poverty mutually reinforce vulnerability.

*A12 (female*,* 16 years old): That night*,* he [her father] came home drunk*,* aggressive*,* what’s the word? Violent*,* and he opened the door*,* or well*,* we saw that he was drunk because he also insulted my mum over the phone for no reason […] he came drunk*,* forced the door open*,* slammed it hard*,* broke it. I mean*,* it was a whole mess. I started screaming*,* my mum started screaming*,* and my dad started hitting her. When I started screaming*,* my grandparents woke up and called the police. My dad […] was a total piece of shit. He was about to hit my grandad. So*,* it was all really horrible*,* and it affected us a lot. […] I remember one time*,* out of nowhere*,* my mum and I had nothing to eat. I mean*,* we didn’t even have anywhere to live*,* like*,* nothing to sleep on.*

#### Sexual orientation

There were no accounts of adolescents voluntarily disclosing their lesbian or bisexual orientation to their families. In cases where families were aware, this knowledge resulted from breaches of privacy, rather than open communication. Family reactions, in all instances, were embedded in heteronormative social scripts, where rejection was often framed as a ‘failure’ of the adolescent to meet traditional family expectations.

*A20 (female*,* 14 years old): When my mum found out*,* she told me I couldn’t be like that*,* that it was a mistake*,* that I was confused. She said I was her only daughter*,* how could I turn out like that?*

#### Appearance

Narratives indicate that adolescents have experienced violence, discrimination, and ridicule from peers, teachers, and the wider community for not conforming to dominant beauty standards. Discriminatory experiences were reported, for example, towards individuals with high body weight or those with smaller body sizes, as well as criticism towards girls whose clothing deviated from traditional, conservative social norms.

*Observation note (28.10.2022): While we were settling in*,* the coordinator of the activity asked us to shift a bit so that everyone could fit. At that moment*,* the (male) teacher said loudly*,* “The thing is*,* there are a few chubby girls.” I noticed some of the students looked at him with annoyance.*

#### Disability status

Another theme is the discrimination, ridicule, and difficulties faced by individuals with disabilities, situations they have experienced directly. Furthermore, the presence of disability within a context of poverty demanded increased care and resources, yet often coincided with the absence of essential material conditions, such as stable housing and adequate food, thereby illustrating how disability and socioeconomic marginalisation mutually constitute a state of extreme vulnerability.

*C3 (female*,* special educator): We are not all in the same circumstances*,* and we don’t all learn the same way. What’s really missing here is a public policy that supports the kids [children with disabilities]. […] In this district*,* there are many people with disabilities*,* but there are no job opportunities*,* no training opportunities*,* no pathway where you can say*,* it is right for the kids.*

In public schools in Bogotá, students with disabilities are often enrolled in mainstream classrooms. Adolescents were aware of the limited living conditions and opportunities experienced by this population. As a result, and within their means, they often made efforts to include, support, and accompany their peers with disabilities.

#### Age

Participants reported that their opinions were often disregarded by parents, describing experiences in which they felt ignored or belittled by adults in their environment. They also noted that their wishes were sometimes overlooked. This adult-centric power dynamic often intersected with gender, as girls’ time was specifically commodified to sustain the household’s care economy. For many girls, domestic responsibilities were not intended as educational but served to reduce the burden on parents. These tasks, ranging from maintaining the household to caring for younger siblings or assisting in their mothers’ informal childcare work, functioned as a crucial but invisible pillar of the family’s socioeconomic survival.

*A19 (male*,* 12 years old): Some of us chose to go to church on Saturdays and others chose to stay at home […] one time I told my mum that I wanted to stay home and rest*,* and she started telling me off because it’s no longer our [the children’s] decision whether we want to go or not; I mean*,* we have to go*,* but I didn’t want to because I was really sleepy*,* I was very tired.*

#### Knowledge and skills

Another form of discrimination identified by the adolescents relates to knowledge and skills. They reported that the possession or lack of certain abilities often became a criterion for inclusion or exclusion within peer groups. Those perceived as more knowledgeable on a given topic tended to be regarded as superior, while those who struggled to understand the subject matter were often met with disdain or ridicule, regardless of the underlying circumstances that might have limited their access to such knowledge. Similarly, in sports, physical performance acted as another basis for social inclusion or exclusion.

*A32 (female*,* 13 years old): At school*,* they used to make fun of her [ her sister] because she didn’t know certain things*,* or she couldn’t read very well*,* or stuttered a lot*,* and that’s something she didn’t choose to have […] Last year*,* her classmates were awful*,* I mean*,* they treated her badly*,* and that’s why she ended up repeating the year.*

#### Cultural identity

Participants described situations they had either experienced directly or witnessed in others, in which racialised adolescents were perceived as having lesser value, excluded from games or social interactions, and in some cases subjected to peer violence. In addition, some interviewees reported being targeted by homogenising and derogatory assumptions, as well as negative value judgements based on their skin colour, perceived intelligence, cultural practices, hygiene, and socioeconomic status.

Adolescents’ narratives also included accounts of adolescents born in Venezuela who had been mistreated by classmates, as well as descriptions of the challenges they faced during the migration process, particularly upon arriving in Bogotá. These included the breakdown of support networks, changes in the educational system, cultural dissonance, differences in climate, issues related to residency permits and documentation, and difficulties faced by their parents in securing housing and employment. Colombian adolescents also reported observing and rejecting xenophobic attitudes among their peers. However, some interviewees themselves also expressed sentiments of rejection, reflecting the complex and sometimes contradictory ways in which xenophobia is reproduced.

*A15 (male*,* 14 years old): Last year*,* we had a friend who was Venezuelan*,* he was in our class and he was dark-skinned*,* Black… And they bullied him a lot*,* to the point where it was really awful. I mean*,* he wasn’t alone*,* there were five of us in our friend group*,* but after seeing that they were only picking on him*,* again and again and again*,* it got to the point where the four of us stepped in and told everyone*,* like*,* “Alright*,* that’s enough now*,*” because it’s not fair. Just because he’s a foreigner and has a different skin colour doesn’t mean they have to treat him like that.*

### Feeling inequality

This theme reflected how adolescents emotionally processed and responded to the inequality that affected their lives. These responses were not interpreted as individual clinical pathologies, but rather as the emotional embodiment of injustice; that is, as forms of social suffering and affective experiences that bridged structural barriers and personal realities across three levels: societal, interpersonal, and individual. This perspective allowed for an understanding of how structural inequality was not merely perceived externally but was internalised and experienced as a subjective burden.

#### Societal

Adolescents’ narratives were marked by emotions such as anger, indignation, and dissatisfaction. These sentiments reflect a widespread discontent with a social order perceived as unjust, particularly towards the most marginalised groups, with whom the participants themselves identify. They expressed both rejection and indignation towards social elites, who benefited from privileges denied to them, and towards political leaders, whom they perceived as incapable of promoting inclusive policies or advancing equity and the protection of rights. Simultaneously, they reported feelings of frustration stemming from their own limited capacity to respond effectively to the challenges they witnessed and experienced.

*A25 (male*,* 16 years old): It’s really awful that so many people suffer from poverty and misery. I mean*,* I think everyone should live as equals*,* right? Not with some being the richest and others the poorest*,* no*,* we should all live equally*,* happily. Because*,* let’s be honest*,* those people [people living in conditions of poverty or destitution e.g.*,* homeless people] aren’t happy. Their whole lives*,* sometimes they’re just struggling for a piece of bread. And people also discriminate them a lot*,* they humiliate them in horrible ways. So yeah… it’s just sad*,* it really doesn’t bring me any joy*,* to be honest.*

#### Interpersonal

Interpersonal relations were also affected by social inequality. According to participants, emotional states such as irritability, anger, contempt, dissatisfaction, and distress were commonly experienced by caregivers as a consequence of economic hardship. These emotional responses often led caregivers to behave in ways that were emotionally distant, unresponsive, or even hostile towards adolescents, frequently resulting in family conflict. This dynamic was closely linked to the emotional experiences of the adolescents themselves, who reported feelings of anger, helplessness, anxiety, and sadness arising from their awareness and perception of the stress endured by their caregivers.

*A26 (male*,* 17 years old): Well*,* you see*,* I used to worry about my mum. Because I knew she wasn’t doing well financially*,* so she was always stressed*,* she looked sad*,* she looked desperate. And that makes you feel desperate too*,* it makes you sad. And yes*,* you want to do something*,* you want to help*,* but you can’t*,* because you don’t have anything. I mean*,* those problems*,* they’re solved with money*,* right? And money*,* well… it’s really*,* really hard to get. So yes*,* I mean*,* her desperation sort of rubbed off on me. So yes*,* I felt desperate.*

During the pandemic, experiences of stress, sadness, and despair linked to families’ economic hardship were intensified, particularly in reference to the main caregivers, mothers. As financial scarcity worsened, adolescents became more acutely aware of these difficulties, leading to feelings of anxiety and helplessness due to their inability to contribute to the household. These emotions also fuelled greater dissatisfaction with their living conditions, especially in relation to the quality and quantity of available food.

#### Personal

Adolescents reported experiencing significant emotional distress associated with their exposure to social inequality. A recurrent emotional experience described by participants was a sense of diminished self-worth and feelings of inferiority or difference. These emotions were often triggered by experiences of exclusion from social activities, games, gatherings, or recreational spaces. Exclusion was frequently linked to participants’ socioeconomic status, non-conformity with traditional gender roles, physical appearance, or experiences of xenophobia. As one participant explained, she felt *“a little less somehow*,* for not being like my classmates and not coming from the same place as them” (A36*,* female*,* 13 years old).*

Many participants associated emotional distress with the constraints imposed by financial hardship. The uncertainty linked to unstable economic conditions within their households also gave rise to feelings of anxiety and despair. Some adolescents expressed concern about being perceived as a burden to their families due to limited household resources, which led them to make decisions such as leaving home or entering the workforce prematurely. Those engaged in paid work reported experiencing exhaustion, as their responsibilities significantly reduced the time available for academic activities, leisure, and social interaction with peers.

*A37 (female*,* 17 years old): Sometimes I feel like crap […] yeah*,* like*,* with my mum*,* I feel like she’s carrying everything on her own*,* and I feel like… ugh*,* like I’m a burden*,* you know? And it’s not even that she makes me feel that way*,* it’s just something I feel myself. And that’s why I decided to look for a job*,* yeah*,* kind of to stop feeling like that.*

Several adolescents reported feelings of frustration and resentment arising from their inability to access material goods such as fashionable clothing or footwear, or to participate in desirable activities like travel or social events. These limitations contributed to a sense of social exclusion and a diminished sense of belonging, particularly when peers could access such resources. As one psychologist noted, some adolescents interpret these experiences through a comparative lens, asking *“why did I get this life and not someone else’s?”*, and may become *“caught up in the anger about the context they have to live in*,*”* focusing more on *“why me”* than on *“how or what can I do to get out of this” (C1*,* female*,* psychologist).*

Participants who identified as homo- or bisexual described experiencing emotional pain, disappointment, and psychological distress following parental rejection upon the disclosure or discovery of their sexual orientation. In cases where parents were unaware, adolescents reported deliberately concealing their identity due to fear of rejection or discrimination within the family. Feelings of disconnection from life and, in some cases, suicidal ideation were also mentioned.

*A4 (female*,* 15 years old): She [her mum] found out that I like women and*,* well*,* she didn’t take it well*,* so we had a fight. Not like a physical fight or anything*,* but it was more like… I told her I’d had enough*,* that if she didn’t support me*,* then what was the point in existing? […] When I told her that*,* if she didn’t support me*,* I said I wanted to die and things like that*,* because*,* well*,* if I didn’t have my mum’s support*,* then what was the point in carrying on?*

Some participants expressed concern and anxiety regarding perceived barriers to achieving their aspirations. Professional success, particularly in fields such as music or sport, was seen as contingent upon access to influential social networks, which they felt excluded from. This perception contributed to a sense of hopelessness and limited future prospects.

*A29 (male*,* 14 years old): I want to study law*,* become a judge and a lawyer of the republic. But that degree is expensive*,* and when it comes to focusing on pursuing a career*,* I do want to*,* but the cost is what affects me*,* what’s making me think twice.*

### Becoming a social agent of change

This theme captured the ways in which young people began to see themselves not only as victims of inequality but as active agents of change. Through critical reflection, the creation of dialogue, and community engagement, they sought to transform their realities and those of others around them, thereby becoming social agents of change.

#### Being critical and reflecting

Some adolescents reflected on how they might contribute to alleviating economic hardship within the household. They reported engaging in various actions such as avoiding non-essential purchases, considering leaving home, saving part of their daily school allowance intended for food to buy school supplies or contribute to the household’s basic needs, selling previously gifted personal belongings, completing schoolwork for others in exchange for payment, or selling sweets in class. Despite these challenges, some narratives also included expressions of gratitude. Some adolescents found entering the labour market as the most viable means of supporting their families economically.

Although their expectations are not always met, participants acknowledged the considerable efforts made by their caregivers to provide for their well-being. As one participant explained, although she sometimes wanted certain things, she knew her mother did not have the financial means and therefore tried not to demand anything, adding that she *“really appreciate[s] what I do have”* and thanks her mother *“because she made the effort” (A10*,* female*,* 14 years old).*

Adolescents expressed dissatisfaction and demonstrated the ability to reflect critically on the injustices and various forms of violence they identified within their communities or families, including those they had personally experienced. Notably, some participants demonstrated critical awareness of social and economic systems and a level of socio-political insight by articulating a critique of broader systemic structures, for example, identifying capitalism as a source of social inequality.

*A10 (female*,* 14 years old): I feel like*,* in some way*,* we’re all equal […] we should unite more*,* because no one knows what someone else’s life has been like […] But yes*,* deep down*,* I do want to help the world*,* because sometimes it’s frustrating to see so many people with money and so many others without*,* literally dying out there. So it’s like… capitalism.*

The complex experiences described by adolescents often prompted critical reflection. They questioned the government’s failure to support the most disadvantaged populations or to create social opportunities that offer protection to those facing barriers in accessing healthcare, education, housing, food, and other basic needs. Participants also emphasised the importance of having leaders capable of mobilising communities to demand improved well-being and social justice.

*A8 (female*,* 16 years old): The government should also provide subsidies for people*,* like actually go to the most marginalised areas and carry out surveys […] There are different subsidies available that can really help people. But there are so many people in need who don’t even know about them. So*,* I feel like they [the government] should pay more attention to those kinds of areas*,* go and find out what’s happening there and why*,* for instance*,* if an aid programme was launched*,* why it hasn’t reached them [the people in need]*,* why they’re still in that situation.*

Adolescents actively sought information, drawing not only on the education provided in school but especially through the internet and social media. These platforms offered them content with which they strongly identified, often prompting critical self-reflection and motivating them to abandon behaviours they recognised as having once been part of their own conduct, but which they subsequently rejected for being harmful or violent.

#### Creating opportunities for dialogue and action

Adolescents expressed hope that new ways of thinking and living could be taught, and sought to become disseminators of the knowledge they gain through reflection. On the one hand, they shared this knowledge with peers, encouraging others, through social interaction, to join them in resisting the systems of power that subordinated them. On the other hand, they introduced these conversations within their households, where their ideas were not always welcomed, but they nonetheless planted seeds of reflection that, over time, began to take root.

*A20 (female*,* 14 years old): Well*,* as we said with my grandmother*,* we realized that social inequality*,* hatred*,* and all that stuff isn’t something you’re born with*,* but something you learn. Mostly from experiences at home and all that*,* especially from people who are older*,* from older generations. Because they’ve been taught that way since they were little […] So*,* I think we should try to talk to other people and make them feel confident.*

Participants envisioned educational strategies targeting different age groups. Some proposed initiatives directed at children, with the aim of fostering a new generation committed to sustaining and enhancing spaces free from discrimination. Others highlighted the importance of educating parents and adults more broadly, so they might become sources of support for their children and create home environments free from violence and harmful stereotypes. Additionally, some adolescents saw potential for engagement with older generations, expressing hope that communication, change, and mutual understanding were still possible.

*A2 (female*,* 16 years old): Maybe we could start improving things by beginning with children*,* talking to them about how inequality isn’t right. […] I think it should start with conversations*,* because if we look at it realistically*,* changing the mindset of adults is already very difficult. So*,* we should begin with the newer generations*,* with the little ones*,* because I think it’s easier to try to convince them that this isn’t right*,* and that we can be better in the future.*

Adolescents emphasised the importance of forming relationships grounded in empathy and solidarity. These relationships were based on recognising the needs of others and offering support through respect for difference and the appreciation of each person’s intrinsic human value. They also described collective actions, through which they organised themselves to address the forms of inequality that they perceived as unjust. These efforts were visibly reflected in notes taken during observation of the school hallways, which documented student-led initiatives displayed on bulletin boards addressing the armed conflict in Colombia and recognising its victims, promoting respect for Afro-Colombian identity, and highlighting women’s rights.

*A29 (male*,* 14 years old): With some friends*,* we are currently doing social work*,* so to speak. Like*,* sometimes on Saturdays or so*,* I help my godfather at his workshop*,* and he gives me some money*,* and with that I buy blankets or stuff like that with my friends*,* and we give them to those in need*,* to people we see on the streets*,* give them a blanket or something*,* because you see them all wrapped up […] Also with dogs*,* when we see dogs on the street*,* we buy them packets of food.*

Regarding gender-based and homophobic violence, adolescent girls who had experienced such forms of discrimination described how they chose to defend their decisions and protect their freedom through dialogue with family members and others in the community. They expressed a refusal to continue hiding or lying about who they are; instead, they actively sought possibilities for change in their families’ responses and pursued spaces where they were valued rather than subjected to violence. These accounts were accompanied by those of other adolescents who recognised this issue in society and committed to participating in conversations aimed at fostering respect for the LGBTQ+ community and their rights.

*A33 (female*,* 13 years old): And so*,* it’s like starting to talk to children from a young age*,* because let’s say*,* I asked my brother*,* who told me that I had a girlfriend*,* but he said it to annoy me*,* and he started making comments that a relationship between a woman and a woman is not possible*,* and my mum kind of started to support him*,* and I said to her*,* but why should it be like that if he has to accept anyone else’s preferences*,* whether they like women*,* men*,* or if they want to be transgender or whatever*,* right? It’s like men are given this privilege to feel entitled to judge other people’s choices*,* right?. It just feels so*,* I don’t know*,* sexist… I don’t know.*

#### Questioning government support in times of crisis

The worsening of living conditions for many families during the pandemic and afterwards prompted adolescents to question the functioning of both national and local governments. They believed that precisely during this period of heightened need, support should have been extended to the most marginalised populations, of which they consider themselves a part. However, the absence of such assistance led to a sense of dissatisfaction with the decisions made by those in power. One professional situating these perceptions within a broader context of social mobilisation noted that, rather than extinguishing previous social unrest in Colombia, the pandemic *“generated a glowing ember*,* which remained there and is now reappearing… people are*,* in a way*,* losing their fear*,* and that’s something that cannot be reversed” (C4*,* male*,* psychologist).*

## Discussion

This study aimed to explore how adolescents living in one of Latin America’s most unequal urban settings experience and engage with intersectional forms of inequality, particularly in the context of the COVID-19 pandemic, drawing on direct observations and problem-centred interviews with adolescents and school counsellors in two socioeconomically disadvantaged neighbourhoods in Bogotá, Colombia. Within this analysis, the pandemic is understood primarily as a context that intensified and rendered more visible pre-existing structural inequalities, rather than as a distinct analytical category. The findings highlight how adolescents interpret and make sense of intersecting forms of disadvantage as part of their everyday lives, showing that inequality is experienced not only as an external structural condition but also as a lived and interpreted reality. In doing so, the study contributes by illustrating how intersectionality is experienced and articulated by adolescents themselves, bringing attention to the ways in which social inequality is perceived, felt, and negotiated within their specific socio-spatial context. The thematic analysis yielded three overarching themes: *perceiving inequality*,* feeling inequality*,* and becoming a social agent of change*.

By centring adolescents’ narratives through qualitative methods, this study foregrounds young people as epistemic subjects capable of articulating how structural inequality shapes their everyday lives. The first overarching theme (presented in Fig. 1, upper left-hand bubble) illustrates how adolescents recognise the material and social dimensions of inequality as lived constraints on their present conditions and future opportunities.

Living in socioeconomically disadvantaged contexts operates as a structural constraint that restricts adolescents’ access not only to basic needs, such as food, housing, and healthcare, but also to educational, recreational, and social opportunities that are central to long-term well-being. These experiences reflect broader evidence on precarious living conditions in Bogotá, driven by factors such as precarious employment, informality, and underemployment [50, 51]. Accounts of economically disadvantaged households, often primarily supported by mothers, were prominent across the data, reflecting the feminisation of poverty and women’s structural disadvantages in the labour market, including informal employment, lower incomes, and greater instability that have been widely documented in the general population [14, 52]. Our findings show how these structural dynamics are also present in adolescents’ experiences, indicating the persistence of these inequalities across generations. Such conditions contribute to the reproduction of inequality and marginalisation over time [53].

Food and housing insecurity appear as central concerns in adolescents’ accounts. Food insecurity aligns with national data indicating that one in two Colombian households experiences some degree of food insecurity [54], while housing insecurity reflects substandard rental conditions, overcrowding, and limited access to basic services in marginaised neighbourhoods of Bogotá [55]. Urban segregation further exacerbates these challenges by relegating low-income families to the city’s periphery, restricting access to essential resources and limiting opportunities for rest, leisure, and family life [28, 53, 56, 57]. Although these conditions have been widely documented, our findings bring into focus how they are experienced and made sense of by adolescents in their everyday lives.

These material constraints were closely linked to perceived violations of fundamental rights, particularly the rights to health and education. Participants highlighted financial barriers to accessing mental health services, consistent with national evidence [58], as well as structural obstacles limiting educational aspirations among adolescents from economically disadvantaged backgrounds [59]. These challenges were intensified during the COVID-19 pandemic, when school closures and digital inequalities created new barriers to secondary education, particularly for adolescents lacking internet connectivity and electronic devices [24, 26, 60, 61]. As reflected in participants’ accounts, the pandemic magnified pre-existing inequalities, further compromising adolescents’ well-being and developmental opportunities.

Beyond socioeconomic disparities, the findings highlight how gender-based inequalities further shape adolescents’ lived experiences. Participants’ accounts indicate that gender roles influence access to opportunities, consistent with national data showing that patriarchal norms constrain women’s autonomy and aspirations [18, 62]. In Colombia, unequal distribution of unpaid care work begins early; weekly, girls perform about five hours of such work more than boys [63], limiting their time for education and personal development [14, 17, 63]. This burden negatively affects school attendance and learning outcomes, reinforcing disadvantage [14, 17]. During the COVID-19 lockdown, these inequalities deepened, with female adolescents assuming greater domestic responsibilities, further restricting their opportunities for rest, study, and leisure [64]. These accounts also point to the persistence of gendered stereotypes that prescribe fixed characteristics for women and men from an early age, with women expected to embody sensitivity, tenderness, and fragility, while men are associated with independence, strength, and leadership. Within adolescents’ narratives, these expectations are not only reproduced but also experienced as constraints, shaping unequal opportunities and giving rise to situations of discrimination, particularly for those who do not conform to these prescribed roles. This resonates with research suggesting that such norms contribute to the ongoing reproduction of gendered hierarchies and the normalisation of male dominance [18, 65].

Participants’ narratives revealed that gender inequality is inextricably linked to gender-based violence, as many witnessed or experienced aggression perpetrated by male partners or family members. These accounts reflect a broader national pattern with high documented numbers for non-lethal injuries due to domestic violence and non-lethal intimate partner violence, 86.1% involving female victims in 2023 [14]. The most extreme form, femicide, accounted for 607 cases in the same year [14, 17]. Our findings underscore how adolescent girls are embedded within a patriarchal system that threatens their social position [66].

However, the impact is not limited to women. This study also highlights how patriarchal systems privilege heterosexuality, leading to the targeted discrimination of those who deviate from heteronormative patterns. Experiences of homophobia and stigmatisation were prominent among participants with diverse sexual orientations, consistent with national data showing widespread bullying in schools, family rejection, and community-level violence [67–69]. These findings reinforce the urgent need for inclusive education and protective policies that safeguard LGBTQ+ adolescents’ rights.

Bullying and exclusion related to body image represent processes of aesthetic injustice that limit social recognition and inclusion. This reflects how patriarchal and capitalist systems reinforce rigid aesthetic ideals through hegemonic beauty standards, which are often promoted by media and digital platforms to associate appearance with social value and success [15, 70–72]. This internalised pressure described by the adolescents aligns with national data showing that unattainable beauty standards in Colombia contribute significantly to peer discrimination and diminished adolescent wellbeing [72–74].

The marginalisation of people with disabilities is interpreted here as a profound form of social injustice, particularly in relation to the lack of opportunities within neighbourhood contexts. This observation is consistent with findings from other studies highlighting the structural and social barriers that hinder full participation for this population [75]. The narratives of our participants confirm that in contexts of poverty, these barriers perpetuate exclusion and reinforce discriminatory attitudes that undermine the dignity of individuals with disabilities and their families [76]. Additionally, possession or lack of specific abilities functioned as a basis for social inclusion or exclusion within peer groups, as reflected in adolescents’ accounts. These dynamics reinforce existing social hierarchies and exacerbate feelings of marginalisation among those who are less privileged, thereby contributing further to the reproduction of social inequality [77, 78].

An important dimension of the analysis relates to adolescents’ perception of being subordinated within a society structured largely around adult needs and authority. Everyday acts of disrespect and devaluation, as reflected in participants’ accounts, illustrate how adult-centred dynamics reinforce broader social inequalities by dismissing young people’s capacities and knowledge [79, 80]. This aligns with research indicating that autonomy is often associated with adulthood, while adolescence is framed as immature and less socially valued [81].

Within the broader context of social inequality, racism is interpreted as a significant dimension shaping adolescents’ experiences. Accounts of exclusion and segregation echo previous studies documenting the persistence of negative stereotypes towards Afro-Colombian populations [20, 23, 82]. These dynamics contribute to deep socioeconomic and health disparities: for example, 30.6% of people identifying as Black or Afro-Colombian live in multidimensional poverty, well above the national average [83, 84]. Such patterns point to the ongoing reproduction of structural racism and systemic inequality.

Xenophobia operates as a form of discrimination shaping adolescents’ experiences, compounding broader processes of social exclusion. In recent years, over two million Venezuelans have migrated to Colombia due to political and economic crises [85], yet many face precarious conditions and limited access to basic rights [21, 23, 31, 86]. Accounts within this study reinforce findings that document widespread discrimination against Venezuelan migrants, including verbal abuse, social exclusion, hate speech, and restricted access to opportunities. These populations are often stigmatised as threats to employment and public safety, strengthening exclusionary narratives and deepening inequality [21, 23, 31, 86].

In sum, adolescents demonstrate a sophisticated capacity to perceive the intersectional and structural nature of social inequality, recognising it as a force rooted in all spheres of society and interwoven across their daily spaces. This study contributes to existing research by highlighting that adolescents do not perceive these barriers as isolated incidents but as interlocking systems of oppression that mutually reinforce one another. This cumulative perception of subordination results in compounded disadvantages for those with intersecting marginalised identities [2, 36], illustrating how the socio-spatial hierarchy of Bogotá is clearly mapped within the adolescents’ own social consciousness.

The second overarching theme (Fig. 1, in the upper right-hand circle) moves beyond perception to illustrate how this intersectional inequality is embodied, giving rise to significant emotional challenges. In line with a collective mental health framework, these experiences are analyzed not as individual clinical pathologies, but as social suffering and emotional distress arising from the tension between adolescents’ expectations and the frustrations of their environment. This theme reveals that the impact of inequality is not merely external; it is a subjective burden that influences the internal lives of adolescents, their families, and their communities, highlighting the social production of suffering in contexts of structural neglect.

To begin, a marked sense of concern regarding the socioeconomic and political situation of their local communities and the country more broadly was evident across adolescents’ accounts. Feelings of indignation and dissatisfaction with the national context have also been documented in previous studies, which highlight how Colombian youths increasingly engage in social mobilisation [87, 88]. This brings into focus how young movements emerge from the deep frustration generated by the widespread poverty affecting the majority of the population and the persistent insufficiency of effective governmental responses to address this situation [88–90].

Secondly, it was found that adolescents’ emotional well-being is highly vulnerable to the economic hardships faced by their families. Changes in parental mood resulting from financial strain often translate into feelings of anxiety and concern among adolescents regarding their family’s situation, while also acting as triggers for family conflicts due to heightened tensions within the household. Previous studies have similarly shown that poverty, low income, and poor job quality result in significant stress within families are associated with increased levels of parental stress [91, 92]. This, in turn, negatively affects family functioning by reducing parents’ emotional availability and sensitivity in responding to their children’s affective needs [93–95]. The presence of such stressors has been shown to foster a hostile family environment, eliciting feelings of sadness, rejection, and helplessness among children and adolescents [94–96].

Thirdly, accounts in this study illustrate how various forms of inequality exert a deeply personal influence, shaping their emotions, decision-making, and life expectations. The association between sadness, anxiety, stress, and intersectional inequality identified in this study aligns with previous research linking social inequality among Latin American adolescents to low self-esteem, diminished positive affect, and the production of social suffering [2]. Material deprivation and financial strain have also been associated with both internalising and externalising behaviours [95]. The emotional toll of poverty often stems from the frustration of unmet expectations in comparison to more affluent peers, contributing to feelings of shame and emotional exhaustion [97]. Regarding the COVID-19 pandemic, not only did intersectional inequalities intensify, but adolescents also experienced heightened emotional distress as a result of the severe economic hardships endured by their families. This has been corroborated by other studies, which highlight how distress was amplified by adolescents’ dual role as both observers and direct victims of these difficulties. This dual burden intensified feelings of anxiety, helplessness, and vulnerability during a period of profound uncertainty [25].

According to the interviews, adolescents’ self-esteem and well-being are also negatively impacted by hegemonic beauty standards imposed by society. Young people often evaluate themselves against these socially constructed ideals and may strive to conform to them. As shown in previous studies, those who feel they fall short of these norms, or who face discrimination as a result, are at greater risk of mental health issues, including not only negative self-perception but also the development of eating disorders [72, 74].

Emotional vulnerability is further exacerbated in migration contexts. Research on mental health and migration has documented psychological distress associated with the challenges of the migratory process, including families’ low socioeconomic status, difficulties in securing employment and financial stability, lack of legal status, cultural dissonance, and experiences of discrimination [98], all of which were also reported by migrant adolescents in this study. These impacts extend beyond an individualised clinical focus, undermining adolescents’ sense of purpose, autonomy, and perceived capacity for self-determination [99].

Furthermore, contexts marked by family poverty are a key driver of child and adolescent labour [100]. Financial hardship is experienced as a personal burden, with some adolescents assuming a sense of responsibility to alleviate their families’ economic struggles.This reflects findings from other research showing that children in precarious living conditions are more likely to engage in informal work, often underpaid and lacking legal protection [101]. Both paid and unpaid labour during these formative years carries serious consequences, including school dropout, poor academic outcomes, and negative impacts on mental health, perpetuating cycles of poverty [100, 102].

Experiences of discrimination related to diverse sexual orientations are associated with forms of emotional distress in adolescents’ accounts. This resonates with research highlighting the adverse mental health implications of heteronormativity and heterosexism, including increased emotional stress and poorer academic engagement [68, 103, 104]. These adolescents are at greater risk of suicidal behaviour, often linked to the psychosocial burden of navigating a stigmatised identity [105]. Moreover, persistent homophobic attitudes within families reinforce harmful beliefs and anxiety, subjecting adolescents to ridicule, verbal abuse, and, in some cases, coercive attempts to “correct” their sexuality [69, 106]. These dynamics increase social suffering, internalised stigma, and engagement in risky behaviours, ultimately constraining adolescents’ autonomy, wellbeing, and freedom [68, 103].

Setting goals and actively planning for the future is a key developmental task during adolescence. As demonstrated in this and previous studies, living in contexts marked by social inequality poses a significant challenge in this regard. Adolescents frequently express concern about their ability to achieve their aspirations, as limited access to economic and social capital can hinder the realisation of their life plans and restrict their perceived future opportunities [2, 107].

Taken together, these findings underscore the profound emotional burden that structural and intersectional inequalities exert on adolescents’ lives. Experiences of poverty, discrimination, stigmatisation, and marginalisation, whether based on economic status, sexual orientation, bodily appearance, or migratory background, intersect to shape adolescents’ emotions, self-perception, and mental wellbeing. These findings highlight the need to address the emotional consequences of social inequality through structural responses, while also recognising and supporting adolescents’ lived experiences within their specific social contexts. Finally, intersectional inequality generates not only emotional challenges for adolescents but also fosters the development of critical capacities to confront the power structures that shape their living conditions and overall wellbeing. Through the third overarching theme (bottom bubble in Fig. 1), this study highlights the various actions undertaken by adolescents to evaluate, question, and seek to transform the systems and practices that undermine both their individual and collective wellbeing, demonstrating their agency and capacity for social critique and change.

One contribution of this study lies in exploring how adolescent agency is understood and enacted within contexts of structural inequality. Rather than portraying adolescents solely as passive recipients of disadvantage, the findings highlight how young people actively interpret, resist, and negotiate oppressive social conditions. These forms of agency appear to play a role in how adolescents make sense of adversity and respond to experiences of stigma and marginalisation, suggesting a potential relevance for mental health and wellbeing.

The findings suggest that adolescent agency may be relevant to how mental health is experienced and negotiated collectively. By moving from passive “victims” of inequality to active “agents of change”, some participants describe forms of engagement that can be understood as psychosocial resistance to structural disadvantage. This shift appears not only cognitive, but also relational and embodied: through critical reflection, such as identifying capitalism, state neglect, or discrimination as sources of social suffering, adolescents articulate interpretations that situate distress within broader structural conditions, rather than solely at the individual level. In this sense, agency can be interpreted as a form of symbolic resistance and collective sense-making, with potential implications for how experiences of marginalisation are understood and navigated. The interviews further indicate that adolescents can engage in critical reflection on the social worlds in which they are embedded. This aligns with previous research showing that children and adolescents can play active and critical roles across family, school, political, and community settings, expanding their civic and political capabilities [108]. Rather than being positioned solely as passive recipients of adult decisions, participants’ accounts illustrate how adolescents can act as social actors with agency, forming their own perspectives and, in some cases, contributing to the shaping of their own lives and those of others [109]. Although traditionally subordinated within adult-centred systems, as this and other studies show, adolescents are also able to question hegemonic discourses and express a desire to participate in transformative processes that reshape their relationships with adults [109, 110].

From a rights-based perspective, adolescents’ actions can be understood in relation to practices of participation and everyday forms of democracy. Their efforts to create spaces for dialogue, whether through school-based initiatives addressing Afro-Colombian identity or through everyday acts of resistance within the family, can be interpreted as expressions of their engagement with social participation. These practices challenge adult-centric paradigms that exclude young people from decision-making and point to participation as a meaningful dimension of social inclusion and equity [109, 111]. Importantly, adolescent agency does not always take the form of large-scale or formally organised action; rather, it is often expressed through everyday practices of dialogue, empathy, and resistance to discrimination. Such acts of self-assertion can be seen as contributing to how adolescents affirm their dignity, make sense of their experiences, and navigate processes related to wellbeing and identity development [111, 112].

Adolescents’ accounts point to forms of agency expressed through participation in spaces of dialogue, peer support, and everyday acts of resistance within schools, families, and neighbourhoods. Within these contexts, some adolescents describe ways of questioning discriminatory norms and imagining alternative futures [113]. This resonates with research highlighting that adolescents can exercise voice and forms of autonomy in ways that position them as active participants in their social environments, even within the constraints imposed by structural inequalities [108, 110].

Overall, the findings of this study highlight adolescents as socially situated actors who actively perceive and interpret their social, political, and economic contexts. Participants’ accounts point to a heightened awareness of structural inequalities, which in some cases is expressed through critical reflection and engagement in spaces of dialogue and collective exchange (Fig. 1). These findings suggest that, alongside experiences of vulnerability, adolescence may also involve forms of critical awareness and participation that are shaped within, and constrained by, broader social structures [114, 115].

## Strengths and limitations

This study offers a significant contribution by amplifying the voices of adolescents on issues that are typically examined through adult-centred perspectives. Rather than portraying young people as passive or highly marginalised individuals, this research positions them as social and political actors with the capacity to reflect critically on their lived realities. By foregrounding adolescents’ perspectives, the study challenges dominant narratives that frame youth primarily in terms of vulnerability or deviance, and instead recognises their agency and interpretive capabilities.

Another strength lies in the timing and context of data collection. Fieldwork was conducted during a pivotal moment in recent global history: the year 2021, when schools in Bogotá began reopening after prolonged closures due to the COVID-19 pandemic. This setting provided a unique window into how an already unequal social landscape was further disrupted by the crisis. The pandemic amplified existing structural inequalities and exposed adolescents to intensified socio-economic hardship and psychosocial strain. Collecting data during this period enabled the study to capture the complex and evolving experiences of young people navigating the aftermath of a global emergency within local conditions of entrenched inequality. At the same time, it is important to note that many of these experiences reflect pre-existing structural conditions, which were rendered more visible and acute during this period. As such, while the data capture pandemic-related experiences, it is not always possible to clearly distinguish between inequalities specific to COVID-19 and those that would likely have emerged regardless. Instead, the pandemic functioned to make these inequalities more visible and, in many cases, more acute within adolescents’ everyday lives.

This study has certain limitations that warrant consideration. Although the selected neighbourhoods are marked by poverty and structural inequality, the research was conducted mainly within schools. As a result, the findings may not fully capture the experiences of adolescents in situations of extreme deprivation, including those not enrolled in school (school non-attendance in Bogotá was 12% in 2018 [116]). This schooling-based recruitment may have shaped how agency is expressed in the data, as adolescents in school are more likely to have access to resources that support the articulation of critical perspectives and aspirations for social change. Consequently, the forms of agency identified should be interpreted as situated and may be less visible among adolescents experiencing more severe social exclusion.

Second, ethical and legal requirements necessitated parental consent for minors. While essential for safeguarding, this limited access to certain perspectives, as some adolescents who wished to participate were unable to do so without parental approval. Despite efforts to ensure confidentiality and provide clear information, parental concerns remained a barrier, potentially excluding voices relevant to the study’s aims. However, observation did not require written consent, which broadened the scope of the target population.

Third, although the recruitment strategy actively encouraged participation from adolescents of diverse gender identities and sexual orientations, the final sample was composed entirely of cisgender and predominantly heterosexual adolescents. This enabled a rich exploration of gendered experiences but limited the representation of other identities, potentially narrowing the scope of the intersectional analysis. Nonetheless, perspectives from diverse gender and sexual identities were addressed through interviews with both adolescents and school counsellors, and were also highlighted in the observational data.

Finally, data collection and primary analysis were largely concentrated in a single researcher (JCSC). While this allowed for a deep, focused immersion in the data, it requires explicit attention to the researcher’s situatedness. To ensure analytical trustworthiness, we employed rigorous reflexivity through analytic memos to document interpretative decisions, and engaged in regular, structured discussions with the wider research team to challenge interpretations and explore alternative perspectives. This also means that the analysis reflects a situated and interpretive standpoint, shaped by the researcher’s positionality and reflexive engagement with the data.

## Conclusion

This study highlights how persistent and structural social inequality, rooted in class, gender, sexual orientation, physical appearance, disability status, age, access to valued knowledge, and cultural background, shapes the everyday realities of adolescents in Bogotá. Drawing on adolescents’ narratives, counsellors’ accounts, and direct observation, the findings indicate that young people are not only aware of structural oppression but are able to interpret how intersecting systems of inequality affect their own lives and those of their families. In this sense, intersectional inequality is experienced not as an abstract condition, but as a lived and meaningful reality that shapes how adolescents understand themselves and their social worlds.

The analysis further highlights the emotional dimensions of inequality. Across social, familial, and personal spheres, intersecting forms of disadvantage shape how adolescents experience, regulate, and make sense of their emotions. These emotional processes are embedded in broader structural contexts and appear to play a central role in the construction of identities, aspirations, and social roles. In this sense, inequality operates not only through material deprivation, but also through forms of social suffering that directly affect adolescents’ mental wellbeing.

Moreover, the findings point to adolescents’ capacity for agency in the face of adversity. Participants described engaging in everyday practices of dialogue, peer solidarity, and community involvement through which they resist, reinterpret, and contest dominant power structures. These forms of agency can be understood as psychosocial processes through which adolescents navigate experiences of inequality, enabling them to avoid being positioned solely as victims and instead positioning themselves as rights-bearing social actors capable of imagining and contributing to alternative futures.

Based on these empirical findings, this study shows that adolescents’ experiences of distress are shaped by the intersection of patriarchal, capitalist, and adult-centric power relations. From a practical perspective, these insights point towards the potential value of health equity approaches informed by a collective mental health framework, which may help shift attention away from individualised and depoliticised understandings of distress towards strategies that engage with the social conditions and power relations shaping adolescents’ daily experiences.

Within the specific urban context of Bogotá, the findings indicate the importance of spaces for collective reflection and participation through which adolescents can articulate experiences of social suffering and express forms of agency. While large-scale structural reforms fall beyond the direct scope of this study, the analysis offers a situated empirical basis for considering the involvement of adolescents as active participants in initiatives aimed at addressing the everyday barriers to dignity, equity, and mental wellbeing identified in their accounts.

## Supplementary Information

Below is the link to the electronic supplementary material.

Supplementary Material 1: Additional file 1: Observation guide used for data collection (.pdf)

Supplementary Material 2: Additional file 2: Problem-centred interviews guide for adolescents (.pdf)

Supplementary Material 3: Additional file 3: Problem-centred interviews guide for counsellors (.pdf)

Supplementary Material 4: Additional file 4: Original Spanish-language quotes (.pdf)

## Data Availability

The data generated and analysed during the current study are not publicly available due to confidentiality agreements and ethical restrictions, as study participants did not consent to their data being shared beyond the research team. However, the observation and interview guides used for data collection are provided in Additional Files 1, 2 and 3.
