# Supplementary material for: “Alright, that’s enough now, because it’s not fair”: A qualitative study on realities of intersectional inequality among adolescents from Bogotá, Colombia
Source: Int J Equity Health. 2026 Jun 4;25:143. doi: 10.1186/s12939-026-02902-2 (PMC13235179; doi:10.1186/s12939-026-02902-2)
Supplement: Supplementary file 1 — Supplementary Material 1: Additional file 1: Observation guide used for data collection (.pdf) [file 12939_2026_2902_MOESM1_ESM.pdf]

Additional file 1: Observation guide used for data collection

***"Alright, that's enough now, because it's not fair"*: A qualitative study on realities of intersectional inequality among adolescents from Bogotá, Colombia**

Johanna Carolina Sánchez-Castro, Nelly Esther Caliz Romero, Laura Pilz González, Katherina Heinrichs, Christiane Stock.

This document presents the observation guide used during the fieldwork for this study. The guide was designed to support systematic and ethically responsible field observations in two socioeconomically disadvantaged neighbourhoods in Bogotá. It served to sensitise the research team and ensure a consistent focus on relevant dimensions across multiple observation sessions.

<u>Observation guide:</u>

The researcher must always keep the field diary readily available, ensuring that all observation activities are recorded in a timely and appropriate manner.

Notes should be kept in an organised manner, and their construction should consider the characteristics of the records in Table A.

Table A: Characteristics of the field notes

**Characteristic**	**Definition**
Description of the observed situation	This involves providing an objective account of the context in which the action takes place. It includes brief notes on the relationships and situations of the individuals within the observed setting and everyday life. Speech or conversations may be included, but no personal data should be recorded.
Impressions	These refer to the subjective record of the observations and are important because they help the researcher evaluate and make sense of what is being observed. They include introspective comments about what the researcher sees and experiences, as well as what these experiences mean to them. Therefore, impressions should be recorded for all observations.
Preliminary reflections	These refer to the interpretations that allow the researcher to examine the elements recorded in the two previous sections. They help to explain what has been observed and to highlight why the observations were relevant to the research. In this section, the researcher should begin analysing the elements emerging from the description, potentially using preliminary analytical categories.
Theoretical notes	These involve relating the described situations and interactions to relevant theoretical frameworks for further analysis. This section initiates a theoretical argument aimed at understanding how the observed elements function within the research problem or object of study.
