# Supplementary material for: “Alright, that’s enough now, because it’s not fair”: A qualitative study on realities of intersectional inequality among adolescents from Bogotá, Colombia
Source: Int J Equity Health. 2026 Jun 4;25:143. doi: 10.1186/s12939-026-02902-2 (PMC13235179; doi:10.1186/s12939-026-02902-2)
Supplement: Supplementary file 2 — Supplementary Material 2: Additional file 2: Problem-centred interviews guide for adolescents (.pdf) [file 12939_2026_2902_MOESM2_ESM.pdf]

Additional file 2: Problem-centred interviews guide for adolescents

***"Alright, that's enough now, because it's not fair"*: A qualitative study on realities of intersectional inequality among adolescents from Bogotá, Colombia**

Johanna Carolina Sánchez-Castro, Nelly Esther Caliz Romero, Laura Pilz González, Katherina Heinrichs, Christiane Stock.

This supplementary file includes the semi-structured interview guide used to explore adolescents' perceptions and experiences regarding social inequality, mental health, and of the COVID-19 pandemic control measures. The guide was designed to facilitate open dialogue while ensuring consistency across interviews.

Introduction - presentation:

My name is Carolina Sánchez Castro, and as part of my doctoral thesis at the Institute of Health and Nursing Science, Charité – Universitätsmedizin Berlin (Germany), I would like to explore how social inequalities influence the mental health of adolescents in Bogotá D.C., particularly during the COVID-19 pandemic.

That is why I would like to invite you to use this space to share your thoughts, opinions, and experiences on the topic. Our conversation will last approximately 45 minutes in the form of an interview. I will also ask you some general questions about yourself, such as your age, gender, country and/or region of origin, and your level of schooling.

Please remember that this is a safe space and that there are no "right" or "wrong" answers. Everything you share with me will remain confidential. I'd also like to remind you that your participation is entirely voluntary, which means you can choose to stop answering or leave the interview at any point. If there is any question you'd rather not answer, just let me know, you are not obliged to respond.

Please remember that this interview will be recorded. I'd like to ask if you agree to this and if you allow me to use a voice recorder during our conversation. All your personal information will remain confidential, and I will make sure that none of your responses can be traced back to you. Also, don't be surprised if I take notes during our talk, this is just to help me remember important information and possibly come back to it later. Please don't let it distract you.

Do you have any questions so far?

All this information is also included in the informed consent form, which you and your mother, father, or legal guardian have signed. By signing that document, you confirmed your consent to participate and agreed to the processing of your personal data.

Demographic questions

Before we begin the interview, I will ask you some questions about your personal and family information. I will fill out this information myself on this printed form I have here (Table B).

Table B: Demographic information for participants

**General information for participants**
1. How old are you?
2. What is your gender?
3. What is your sexual orientation?
4. What country and/or region are you from?
5. Which neighbourhood do you live in?
6. What grade are you in?
7. Are you currently involved in or interested in joining any community group? Which one?
8. About the people you live with:

Relationship	Age	Education level	Occupation	Employment Status

9. What pets do you have?
10. What type of family do you have?
11. About your housing:
11.1 Housing ownership
11.2 Housing cohabitation
11.3 Number of bathrooms
11.4 Number of bedrooms
11.5 Internet access (Wi-Fi)

<u>Start of the Interview</u>

(Audio recording begins)

The following questions are related to the objectives of the research and will be asked during the interviews with the participants (Table C):

Table C: Questions for adolescent participants

**Category**	**Questions**
Ice-breaker question	1. Can you tell me about your daily routine on the days you go to school?

Category	Questions
Familiarity with social inequality	2. How familiar are you with the term *social inequality*?<br><br>*Follow-up: If the participant says they are not familiar with the term, a brief explanation will be provided to help them understand it and relate it to their own experiences or knowledge.*<br><br>Based on their answer, you may ask whether the participant knows the concept but refers to it by another name, or whether they have experienced situations in their life that are related to it and that would allow them to continue participating in the interview. The aim is to build a shared understanding of the term *social inequality*, so that both interviewer and participant are on the same page during the conversation.<br><br>Definition of social inequality:<br><br>The definition will be printed and made available so that the participant can read it easily.
Feelings related to social inequality	3. How do you feel about social inequality?<br>3.1. How do you think your wellbeing might be affected by social inequality?
Familiarity with COVID-19 control measures	4. What pandemic control measures have affected your life and that of your family – and how?<br>4.1. How did you feel in the context of these measures?<br><br>*Follow-up: If the participant says they are not familiar with the disease control measures, a brief explanation will be provided to help them become familiar with the topic.*<br><br>Examples of COVID-19 control measures:<br>*These will be printed and available for the participant to view if needed.*
Influence of COVID-19 control measures and social inequality on adolescents' lives	5. What changes occurred in your relationships with family members due to the COVID-19 control measures?<br>6. What personal or behavioural changes have you experienced as a result of the COVID-19 control measures?<br>6.1. What positive effects on your emotions or life do you think the COVID-19 control measures have had on you?<br>6.2. What negative effects on your emotions or life do you think the COVID-19 control measures have had on you?<br>7. How do you think people in your community, your family, or yourself were particularly affected by the pandemic? (e.g. economic changes).

Category	Questions
	8. How do you feel your outlook on life changed because of the pandemic?
	9. In what ways do you think social inequality influenced what you experienced during the COVID-19 pandemic?<br>9.1. What impact did this have on your life?<br>9.2. Can you tell me about your experience when you or someone in your family was ill or needed healthcare services? How do you think these situations were influenced by social inequality?<br>10. How do you think social inequality changed or was influenced by the pandemic?
General aspects of mental health	11. Now I'd like to talk about the kinds of things you think are important for enjoying life. For example, you can tell me about situations when you feel happy or feel good.
Changes in adolescents' mental health related to social inequality and COVID-19 control measures	12. How do you think social inequality affects your ability to enjoy life?
	13. What emotions come up for you when you think about social inequality and COVID-19?
	14. How were your personal relationships with family and/or friends affected by COVID-19 and social inequality?
	15. How do you think your life plans have been affected by social inequality, especially during the pandemic?
	16. Thank you for sharing your experiences with me. I'd like to know, how do you think your situation could be improved?<br>17. How do you think you, or other young people like you, could help improve your situation?
Final	18. Is there anything else you would like to add?
