# Supplementary material for: “Alright, that’s enough now, because it’s not fair”: A qualitative study on realities of intersectional inequality among adolescents from Bogotá, Colombia
Source: Int J Equity Health. 2026 Jun 4;25:143. doi: 10.1186/s12939-026-02902-2 (PMC13235179; doi:10.1186/s12939-026-02902-2)
Supplement: Supplementary file 4 — Supplementary Material 4: Additional file 4: Original Spanish-language quotes (.pdf) [file 12939_2026_2902_MOESM4_ESM.pdf]

Additional file 4: Original Spanish-language quotes

***"Alright, that's enough now, because it's not fair"*: A qualitative study on realities of intersectional inequality among adolescents from Bogotá, Colombia**

Johanna Carolina Sánchez-Castro, Nelly Esther Caliz Romero, Laura Pilz González, Katherina Heinrichs, Christiane Stock.

This supplementary file presents the original Spanish-language quotes corresponding to the translated excerpts included in the main article (Table E). The quotes are listed using the same identification codes as in the article. They are presented in the order in which they appear in the text to ensure traceability and transparency in the translation process.

Table E: Translated and original quotes

Quote ID	English Version (as published)	Original Spanish Version
**A12**	Right now, we're not doing too well. My mum, well, she got a slight raise, but it's still not enough because we have to pay for, let's say, the flat, […] the service charge. We also have to pay for food and utilities […] food is very expensive, too expensive. I mean, you see something and think, "Oh, that's quite cheap," but when you add everything up, it's extremely expensive. My mum... right now, we don't have a great financial situation to buy loads of food, like a really big purchase […]. We don't have much to eat, but we're not really struggling or starving, because my brother's godparents live here too [godparents are the social and economic support network for the adolescent's family].	Actualmente no nos vemos muy bien. Mi mamá pues ya le subieron un poco el sueldo, pero igual no alcanza porque tenemos que pagar como digamos el apartamento [...] la administración [...] tenemos que pagar alimentos y los servicios [...] la comida es muy cara, demasiado cara. O sea, yo veo o sea tú ves una cosa y tú dices, ah está muy barato, pero tú juntas todo y es extremadamente caro. Mi mamá, nosotros en este momento no es que tengamos muy buena economía para estar comprando comida, o sea mercado extra grande [...] no tenemos casi que comer, pero pues no nos vemos afectados ni nos estamos muriendo de hambre, porque en ese conjunto vive los padrinos de mi hermano [los padrinos son la red de apoyo social y económico para la familia de la adolescente].
**A19**	It's not actually a flat; it's a commercial space where we live, but we haven't been able to move out because we just don't have enough money.	es que no es un apartamento, es un local, en el que nosotros vivimos; pero nosotros no nos hemos podido ir de ahí porque no nos alcanza la plata.

Quote ID	English Version (as published)	Original Spanish Version
**A2**	I usually wake up at four in the morning because I live in Kennedy [Kennedy and Bosa are districts of Bogotá which, although adjacent, may present challenges in terms of mobility and public transport connectivity between them], so it’s quite a long journey. I walk with my brother because, personally, I don’t like public transport; it’s really difficult to catch and get there on time, so we walk instead. I also wake up early to help my mum get things ready for work because she leaves at around 5:30, so I make sure her breakfast is ready.	Normalmente me levanto a la cuatro de la mañana porque vivo en Kennedy [Kennedy y Bosa son distritos de Bogotá que, aunque adyacentes, pueden presentar retos en términos de movilidad y conectividad de transporte público entre ellos], entonces es un trascurso largo, me vengo caminando con mi hermano porque personalmente no me gusta el transporte público, es muy difícil cogerlo y alcanzar a llegar a tiempo, entonces nos venimos caminando. Me levanto temprano también para ayudarle a mi mami como a alistar las cosas del trabajo porque pues ella parte a las 5:30 más o menos, entonces le dejo como el desayuno listo.
**A42**	I went to the doctor. They gave me some sleeping pills and referred me to a psychologist, but they scheduled my appointment for three months later. So, it didn’t really help much, you could say [...] I was still feeling low, and I was just waiting for the psychologist’s appointment so he could sit down and tell me, “Look…” So, I went, but it didn’t help much, because it was the first time, and it was more like, "What’s your name?" and that was it. "We’ll have a proper chat next time."	al médico sí, me dieron unas pastas para dormir, me mandaron con el psicólogo, pero el psicólogo me lo dieron, la cita me la dieron tres meses después. Entonces no sirvió de a mucho puede decirse […] pues yo todavía estaba mal y yo estaba esperando, era la cita del psicólogo para que él se sentará en me dijera, mira… entonces yo fui, no me ayudó tanto puede decirse porque era una primera vez y era como que tu nombre y eso y ya para la próxima hablamos bien, pero ¿qué pasó?, pero en 5 palabras, ¿qué pasó?.
**A8**	when it comes to getting into university, a person with resources can easily enrol in a professional degree programme, like medicine, and start their studies straight away. Meanwhile, I have to look for scholarships, figure out which university I	Ahorita en entrar a la Universidad, una persona con recursos puede entrar a la carrera, por ejemplo, a medicina, puede entrar muy fácil, puede ya iniciar sus estudios, mientras tanto que ya ahorita tengo que ver becas, tengo que

Quote ID	English Version (as published)	Original Spanish Version
	can afford, so I don't end up in debt, for example, with a loan, because that could really stress me out. So, it's that, it directly affects me, because not having the right socio-economic background means I can't fully meet my needs.	empezar a ver a qué Universidad puedo entrar que me alcance, que pueda yo pagar para tampoco terminar endeudada, por ejemplo, con un crédito, porque eso también me puede mortificar bastante. Entonces es como eso, que, sí me afecta directamente, que pues por no tener el estrato socioeconómico, no puedo cumplir del todo mis necesidades.
**C1**	A student described it to me like this:, "Teacher, I realised I was poor when the pandemic hit, because before that, I hadn't realised I was poor, because I went to school and there I was distracted with my classmates, they [the school] gave me food there, I got uniforms from donations and stuff. But being stuck at home, there was no food. My mom lost her job, my dad lost his job, everyone was just there, at home" [...] so, from there, you can really see how the system of inequality and unfairness works.	Un chico me lo refería así puntualmente y me decía "profe es que yo me di cuenta que era pobre cuando llegó pandemia, porque antes de eso yo no me he dado cuenta que era pobre pues yo iba al colegio y allá me distraía con mis compañeros, me daban la comida, eh pues me daban los uniformes de los que donaban y todo eso, pero estando en la casa pues no había que comer, mi mamá se quedó sin trabajo, mi papá se quedó sin trabajo, todos ahí en la casa" [...] entonces desde ahí se evidencia como el sistema de inequidad, de desigualdad.
**A24**	Oh yes, there was definitely a time when we really struggled, because my mom had to borrow money from different people just so we could eat. Same with my stepdad because he had lost his job. So there was a time when all we ate was like rice with eggs, potatoes, things like that. But later on things started getting a bit better. Still, when we were struggling with money, it was really hard, to be honest.	Uy sí hubo un momento en el que sufrimos mucho, porque a mi mamá le tocó pedir prestado en varias partes para poder comer. Lo mismo mi padrastro porque se había quedado sin trabajo, entonces sí hubo un tiempo el que solamente se comía como arroz con huevo, papas, cosas así, pero ya después la situación fue mejorando, pero cuando sufrimos por plata sí fue duro la verdad.
**A37**	In fact, we had to move somewhere cheaper, smaller, and kind of ugly,	De hecho que nos tuvimos que mudar algo más económico y más pequeño y

Quote ID	English Version (as published)	Original Spanish Version
	because it’s like a little hole, you know? So that really affected us a lot	como feo, porque eso es como en un huequito ¿sí?, entonces eso nos afectó mucho.
**A8**	We were going to call an ambulance to come for him, because he couldn’t breathe […] and the emergency services didn’t even answer us, the phone didn’t even ring. I mean, we tried. So my dad said, no, I’m going to the hospital, and he had to go on his own, walking.	Nosotros íbamos a llamar una ambulancia para que viniera por él, porque él no podía respirar, él no podía, o sea, se paraba, tenía que ir a la ventana a respirar. Y los servicios de llamar [a la ambulancia] ni siquiera nos contestaron, ni siquiera sonaba el tono. Entonces mi papá dijo, no, yo me voy al hospital y él se fue al hospital, solo se tuvo que ir caminando.
**C1**	A lot of the kids from here [the eschool where she works] and at other institutions didn’t have access to internet, or they didn’t have electricity, they didn’t have a phone or a computer where they could, like, do their assignments. So I think that’s where the issue of inequality became a bit more pronounced, because before the lockdown, when they would come here [to the school], they all had the same teacher in the same classroom. Sure, not everyone had the same resources, but the idea was to give everyone the same opportunities. Due to the pandemic, it became very noticeable, because not everyone had access to the tools they needed to access education as such, the education provided by the school.	Muchos de los chicos de acá y pues de otras instituciones no tenían acceso a Internet o no tenían servicio de electricidad, no tenían un celular, no tenían un computador donde poder, pues donde hacer las tareas, entonces ellos desde ahí yo creo que sí se marcó un poquito más como este tema de desigualdad a diferencia, pues antes de la cuarentena que si venían acá, todos pues estaban con el mismo profesor en las mismas aulas, si bien no tenían los mismos recursos todos, pero pues digamos que se les trataba como dar lo mismo a todos. A raíz de la pandemia sí pues se notó demasiado por lo que tengo, porque no todos tenían acceso como a los medios para acceder, pues a la educación como tal, a la educación que les brindaba el colegio.
**A42**	Lately, it’s always like, "Oh, poor him, he needs this," or "poor thing, he has to do that because he works." Like, "Oh, we need to let him rest", or "we need to have	Últimamente es como que “su hermano, pobrecito, necesita tal cosa” o “pobrecito, tiene que hacer éste por lo que trabaja” “toca dejarlo descansar” o

Quote ID	English Version (as published)	Original Spanish Version
	his food ready." For example, my brother never does any housework, he never even picks up a broom, but my granny and my mum do because they are women. I have to pick up a broom and do the chores, and I can't do what they [male family members] do, just go out and leave everything messy, or leave a plate of food lying around. You just can't do that. And they've even said it to my face many times: "But he's a man, and you're a woman, and women do this." So ...	"toca tenerle la comida". Por ejemplo, ellos, mi hermano nunca hace oficio en la casa, nunca coge una escoba, pero mi abuelita y mi mamá sí por ser mujer, sí tengo que coger una escoba para hacer oficio y no puedo hacer lo mismo de ellos de salir y dejo todo ahí desarreglado, o les dejo el plato de comida ahí botado. Entonces uno no puede hacer eso y muchas veces me lo dijeron en mi cara "pero es que él es hombre, entonces usted es una mujer y la mujer hace esto" entonces…
**A12**	That had a big influence on my life here at school, because they [classmates] used to call me "marimacha" [Spanish for tomboy], like, "the boys will see you," and yeah, it was true, but still, sometimes they would exclude me in such a horrible way, and I hate being excluded, or seeing someone else being excluded, because I know exactly how that feels.	Eso influyó tanto en mi vida acá en el colegio porque me decían marimacha, que te vean los chinos y sí, era verdad, pero pues eso a veces me excluían feísimo y odio cuando me excluyen o excluyen a otra persona, porque yo sé cómo se siente eso.
**A12**	That night, he [her father] came home drunk, aggressive, what's the word? Violent, and he opened the door, or well, we saw that ha was drunk because he also insulted my mum over the phone for no reason […] he came drunk, forced the door open, slammed it hard, broke it. I mean, it was a whole mess. I started screaming, my mum started screaming, and my dad started hitting her. When I started screaming, my grandparents woke up and called the police. My dad [...] was a total piece of shit. He was about to hit my grandad. So, it was all really horrible, and it affected us a lot. [...] I remember one time, out of nowhere, my mum and I had nothing to eat. I mean, we didn't even have anywhere to live, like, nothing to sleep on.	esa noche él llegó, borracho llegó brusco, ¿cómo se llama eso? violento y él abrió la, o bueno, como vimos que llegó borracho, porque aparte también insultó a mi mamá en una llamada de la nada […] él llegó borracho, llegó, abrió, golpeó re duro, rompió la puerta. O sea, eso un poco de cosas. Yo empecé a gritar, mi mamá empezó a gritar, mi papá empezó a pegarle. Ehhh yo como empecé a gritar mis abuelos se despertaron y llamaron a la policía. Mi papá […] una mierda completa. Entonces él le iba a pegar a mi abuelo, estaba en pose de que le iba a pegar. Entonces todo fue muy feo, eso afectó mucho […] yo me acuerdo que una vez de la nada mi mamá y yo no teníamos

Quote ID	English Version (as published)	Original Spanish Version
		nada que comer, no tenía o sea, no teníamos ni donde vivir, o sea, cosas en donde poder dormir.
**A20**	When my mum found out, she told me I couldn't be like that, that it was a mistake, that I was confused. She said I was her only daughter, how could I turn out like that?	pues mi mamá cuando se enteró, me dijo que yo no podía ser así que eso era un error, que era confusión, que yo era su única hija, que cómo le iba a salir así.
**Observation note (28.10.2022):**	While we were settling in, the coordinator of the activity asked us to shift a bit so that everyone could fit. At that moment, the (male) teacher said loudly, "The thing is, there are a few chubby girls." I noticed some of the students looked at him with annoyance.	Cuando nos estábamos acomodando la coordinadora de la actividad dijo que nos corriéramos un poco para que cupiéramos todos, el profesor en ese momento dijo en voz alta "lo que pasa es que hay unas cuantas niñas gorditas" vi como algunas estudiantes lo miraron con molestia.
**O3**	We are not all in the same circumstances, and we don't all learn the same way. What's really missing here is a public policy that supports the kids [children with disabilities]. [...] In this district, there are many people with disabilities, but there are no job opportunities, no training opportunities, no pathway where you can say, it is right for the kids	Todos no estamos en las mismas condiciones, todos no aprendemos igual, acá, algo que falta mucho es una política pública que ayude a los chicos [...] acá en la localidad hay mucha gente en condición de discapacidad, que no hay una oferta laboral, no hay una oferta de formación, no hay una oferta donde pueda decir uno, los chicos van a ir bien por este lado.
**A19**	Some of us chose to go to church on Saturdays and others chose to stay at home […] one time I told my mum that I wanted to stay home and rest, and she started telling me off because it's no longer our [the children's] decision whether we want to go or not; I mean, we have to go,	uno escogió que…. que los sábados vayan a la iglesia y otros escogieron que los sábados se queden en la casa, entonces no estén de acuerdo [...] una vez que le dije a mi mamá que me quería quedar en la casa descansando, y me empezó a regañar porque ya no es decisión nuestra si queremos ir o no;

Quote ID	English Version (as published)	Original Spanish Version
	but I didn’t want to because I was really sleepy, I was very tired.	o sea tenemos que ir, pero yo no quería ir porque tenía mucho sueño, estaba muy cansado.
**A32**	At school, they used to make fun of her [her sister] because she didn’t know certain things, or she couldn’t read very well, or stuttered a lot, and that’s something she didn’t choose to have [...] Last year, her classmates were awful, I mean, they treated her badly, and that’s why she ended up repeating the year, because she didn’t know how to read and all that. And one day during the holidays, she was really down, just kind of switched off, and someone asked her what was wrong, and she just started crying, saying they used to make fun of her.	Porque a ella [la hermana] en el colegio como que se burlaban de ella porque como que no sabía ciertas cosas o no sabía leer bien o se trababa mucho y es algo que ella no decidió tener, que no decidió que le tocara […] el anterior año sus compañeros eran terribles, o sea, la trataron mal y por eso como que ella perdió un año, o sea perdió ese por no saber leer y todo eso. Y como que le preguntaron un día como que en vacaciones, ella estaba como que toda triste, como que ahí estaba apagada y como que le preguntaron ¿qué pasó? Y ella como que comenzó a llorar, que se burlaban de ella.
**A15**	Last year, we had a friend who was Venezuelan, he was in our class and he was dark-skinned, Black... And they bullied him a lot, to the point where it was really awful. I mean, he wasn’t alone, there were five of us in our friend group, but after seeing that they were only picking on him, again and again and again, it got to the point where the four of us stepped in and told everyone, like, “Alright, that’s enough now,” because it’s not fair. Just because he’s a foreigner and has a different skin colour doesn’t mean they have to treat him like that.	El año pasado nosotros teníamos un amigo que era venezolano, que era del salón y que era morenito, negrito... Y a él lo molestaban demasiado, pero a un punto en que el bulliying era terrible, o sea, él no estaba sólo, éramos nuestro grupo de amigos de cinco ahí, pero entonces, ya de tanto ver que sólo lo molestaban a él y sólo a él, y sólo a él, y sólo a él, llegó un punto en que ya los cuatro que estábamos con él ya nos metimos a decirle a todos como que bueno ya párenla, porque ya o sea, no es justo porque simplemente porque sea extranjero y de otro color no significa que le tengan que hacer ese tipo de molestias.

Quote ID	English Version (as published)	Original Spanish Version
**A25**	Ugh, well... it's sad. It's really awful that so many people suffer from poverty and misery. I mean, I think everyone should live as equals, right? Not with some being the richest and others the poorest, no, we should all live equally, happily. Because, let's be honest, those people [people living in conditions of poverty or destitution e.g., homeless people] aren't happy. Their whole lives, sometimes they're just struggling for a piece of bread. And people also discriminate them a lot, they humiliate them in horrible ways. So yeah... it's just sad, it really doesn't bring me any joy, to be honest.	uich, pues triste, es muy feo que, pues tanta gente sufra de pobreza, de miseria. Pues todo mundo, digo yo, deberíamos vivir como iguales, ¿No? Tampoco que el más rico y el más pobre, no, pues deberíamos vivir todos igual, felices, porque digamos, esa gente [personas viviendo en pobreza extrema o miseria, por ejemplo habitantes de calle] no es feliz. Toda su vida, a veces luchando por un pedazo de pan; y la gente también los discrimina mucho, los humilla muy feo. Y pues sí, triste, no me da emoción la verdad.
**A26**	Well, you see, I used to worry about my mum. Because I knew she wasn't doing well financially, so she was always stressed, she looked sad, she looked desperate. And that makes you feel desperate too, it makes you sad. And yes, you want to do something, you want to help, but you can't, because you don't have anything. I mean, those problems, they're solved with money, right? And money, well... it's really, really hard to get. So yes, I mean, her desperation sort of rubbed off on me. So yes, I felt desperate.	pues mira que yo me preocupaba por mi mamá. Porque pues yo sabía que ella no estaba bien económicamente, entonces ella se vivía estresada, se veía así triste, se veía desesperada. Entonces eso a uno lo desespera también, lo pone triste. Y sí, como que uno quiere hacer cosas, uno como cómo te ayudo, pero uno no puede ayudar porque uno no tiene, o sea, esos problemas se solucionas es con plata, cierto, entonces, uno plata muy, muy difícil conseguir, entonces sí, o sea como el desespero, ella me lo pegaba a mí. Entonces sí me sentía desesperado.
**A39**	My mom would get stressed out, sometimes she would cry because of it [economic hardship], especially when she couldn't pay the rent on time. […] That made me feel kind of anxious, because	Mi mamá se estresaba, mi mamá a veces lloraba por eso [dificultades económicas] y que pues a veces por no pagar el arriendo a tiempo […] pues me angustiaba un poquito, porque ella

Quote ID	English Version (as published)	Original Spanish Version
	she was clearly worried, even though she kept saying everything was fine. But you could see it was because of that […] I felt kind of sad for my mom, because seeing her cry like that, it hit me hard, yeah, 'cause she's my mom.	estaba preocupada, pero ella decía que no, que no tenía nada, pero pues a ella se le notaba que pues era por eso […] yo me sentía como un poquito triste por mi mamá, porque pues ver a mi mamá llorando así, me ha duro, sí, porque pues es mi mamá.
**A36**	Well, I don't know, it's just that I feel a little less somehow, for not being like my classmates and not coming from the same place as them.	Pues no sé, es que yo me siento un poquito menos por no ser como mis compañeros, y no venir del mismo sitio que ellos.
**A37**	sometimes I feel like crap […] yeah, like, with my mum, I feel like she's carrying everything on her own, and I feel like… ugh, like I'm a burden, you know? And it's not even that she makes me feel that way, it's just something I feel myself. And that's why I decided to look for a job, yeah, kind of to stop feeling like that.	a veces me siento como una caca […] sí, o sea como como que sí con mi mamá como yo siento que ella va con todo sola, me siento como ashh, o sea como una carga ¿sí? y ella no me hace sentir eso, sino que yo lo siento sola y por eso ¿cómo te digo?, tomé la decisión de buscar trabajar, sí como que para no sentirme así
**C1**	They [students] look at it more from a "why did I get this life and not someone else's?" kind of perspective. Some kids really focus on that, they get caught up in the anger about the context they have to live in. Even though they do want to get out of that situation, they focus more on the "why me, why me, why me?" rather than on "how or what can I do to get out of this?"	[los estudiantes] como que lo miran más desde el ¿por qué a mí me tocó esta vida y no me tocó la vida del otro? Hay chicos que lo enfocan mucho como en ese como en ese se guían a veces mucho por la rabia del contexto que les toca vivir. Sabiendo que, pues quieren salir de ese contexto, pero se enfocan más como en ¿por qué a mí, porque a mí, porque a mí?, más allá de ¿cómo o qué puedo hacer para salir de ese contexto?
**A4**	She [her mum] found out that I like women and, well, she didn't take it well, so we had a fight. Not like a physical fight or anything,	ella [la mamá] se enteró que me gustaban las mujeres y pues como que no le gustó y pues y sí tuvimos una

Quote ID	English Version (as published)	Original Spanish Version
	but it was more like... I told her I'd had enough, that if she didn't support me, then what was the point in existing? [...] When I told her that, if she didn't support me, I said I wanted to die and things like that, because, well, if I didn't have my mum's support, then what was the point in carrying on?	pelea, pues no pelea así como de pegarnos o cosas así, pero pues sí era como de… yo le dije que ya no más, que si ella no me apoyaba yo para qué iba a seguir existiendo […] cuando le dije pues que, si ella no me apoyaba que yo me quería morir y cosas así, porque pues si no tenía el apoyo de mi mamá para que iba a seguir existiendo.
**A29**	I want to study law, become a judge and a lawyer of the republic. But that degree is expensive, and when it comes to focusing on pursuing a career, I do want to, but the cost is what affects me, what's making me think twice. My dad tells me not to worry about that, that maybe by the time I finish school there'll be more support or something, maybe he'll be in a better financial position. So, we should wait and see what life brings us.	yo quiero estudiar derecho, derecho, ser juez y abogado de la república. Entonces, pues esa carrera es cara y digamos, lo del enfoque de darme una carrera es sí quiero, pero el costo es lo que me afecta, lo que me está poniendo a pensar. Mi papá me dice que no piense en eso, que de aquí a que salga de estudiar, de pronto hay más ayudas o algo, de pronto él tenga una mejor economía, pues esperar qué nos da la vida.
**A10**	I say like, I'd like to have something like this, but I can't, because sometimes my mum doesn't have the financial means. But I really appreciate what I do have. So, for example, if my mum gives me something similar, I say, oh thank you so much, thanks because she made the effort and all that. And I really value it, like when my mum doesn't have money, I tell her not to worry, because I'm not going to be demanding anything from her.	digo como me gustaría tener tipo esto, pero no puedo, porque a veces mi mamá no tiene como el poder adquisitivo, pero valoro mucho lo que tengo, entonces digamos que mi mamá me da algo parecido y yo digo, ah muchísimas gracias, gracias porque se esforzó y eso y además valoro mucho como digamos a veces que mi mamá no tiene dinero y yo le digo mami que no se preocupe, porque yo no voy a estarle exigiendo.
**A10**	I feel like, in some way, we're all equal [...] we should unite more, because no one knows what someone else's life has been like [...] But yes, deep down, I do want to help the world, because sometimes it's	El Gobierno debería poner también subsidios para las personas, o sea ir a los lugares más marginados y hacer encuestas […] ofrecen diferentes subsidios que pueden ayudar a las

Quote ID	English Version (as published)	Original Spanish Version
	frustrating to see so many people with money and so many others without, literally dying out there. So it's like... capitalism.	personas, pero hay muchas personas necesitadas que no saben ni siquiera. Entonces, yo siento que deberían poner más atención a ese tipo de zonas, ir a conocer qué pasa allá y por qué, por ejemplo, si hicieron una ayuda porque no les ha llegado, por qué siguen en esa situación.
**A8**	The government should also provide subsidies for people, like actually go to the most marginalised areas and carry out surveys [...] There are different subsidies available that can really help people. But there are so many people in need who don't even know about them. So, I feel like they [the government] should pay more attention to those kinds of areas, go and find out what's happening there and why, for instance, if an aid programme was launched, why it hasn't reached them [the people in need], why they're still in that situation.	El Gobierno debería poner también subsidios para las personas, o sea ir a los lugares más marginados y hacer encuestas […] ofrecen diferentes subsidios que pueden ayudar a las personas, pero hay muchas personas necesitadas que no saben ni siquiera. Entonces, yo siento que deberían poner más atención a ese tipo de zonas, ir a conocer qué pasa allá y por qué, por ejemplo, si hicieron una ayuda porque no les ha llegado, por qué siguen en esa situación.
**A20**	Well, as we said with my grandmother, we realized that social inequality, hatred, and all that stuff isn't something you're born with, but something you learn. Mostly from experiences at home and all that, especially from people who are older, from older generations. Because they've been taught that way since they were little […] So, I think we should try to talk to other people and make them feel confident.	pues como dijimos con mi abuela, de que nos dimos cuenta de que la desigualdad social, el odio y todo eso, no es algo con lo que se nace, sino que se aprende. Más que todo con vivencias en casa y eso, más que todo las personas que son mayores, de las antiguas generaciones. Porque ellos desde pequeño las le han metido, pues un modo de crianza un poco diferente. Entonces yo creo que deberíamos de intentar hablar con las demás personas, y hacerles tener confianza.
**A2**	Maybe we could start improving things by beginning with children, talking to them about how inequality isn't right. […] I think it should start with conversations, because	Tal vez se podría empezar a mejorar empezando por los niños, hablándoles de que la desigualdad no está bien […] creo que empezar por charlas ya que si

Quote ID	English Version (as published)	Original Spanish Version
	if we look at it realistically, changing the mindset of adults is already very difficult. So, we should begin with the newer generations, with the little ones, because I think it's easier to try to convince them that this isn't right, and that we can be better in the future.	lo vemos los adultos para cambiar su mentalidad ya sería muy difícil, entonces empezar por con las nuevas generaciones, con los pequeños que creo que sería más fácil empezar a tratar de convencerlos a ellos de que eso no está bien, y que podemos ser mejores en el futuro.
**Observation note (01.11.2022):**	I walked through several hallways in the school and saw that there are various bulletin boards made by students on specific topics: the armed conflict in Colombia and the recognition of its victims, respect for Afro-Colombian identity, and women's rights.	He caminado por varios pasillos del colegio, y he encontrado que hay varias carteleras hechas por los estudiantes sobre unos temas específicos: el conflicto armado en Colombia y el reconocimiento de las víctimas de ese conflicto, el respeto por la afrocolombianidad, y los derechos de las mujeres.
**A29**	With some friends, we are currently doing social work, so to speak. Like, sometimes on Saturdays or so, I help my godfather at his workshop, and he gives me some money, and with that I buy blankets or stuff like that with my friends, and we give them to those in need, to people we see on the streets, give them a blanket or something, because you see them all wrapped up [...] Also with dogs, when we see dogs on the street, we buy them packets of food.	con unos amigos, estamos haciendo ahorita una labor social de que digamos nosotros de parte, yo a veces los sábados o algo, le ayudo a mi padrino en el taller que tiene allí, y él me da plata y pues yo compro cobijas o algo con mis amigos, y ahí vamos dándole a los que necesitan, a los que vemos en la calle, su cobija o algo, porque pues uno los ve así tapados […] también con los perros, vemos perros en la calle, pues compramos paquetes de comida.
**A33**	And so, it's like starting to talk to children from a young age, because let's say, I asked my brother, who told me that I had a girlfriend, but he said it to annoy me, and he started making comments that a relationship between a woman and a woman is not possible, and my mum kind	y o sea es como empezar a hablarles a los niños desde niños, porque, digamos la otra vez, le pregunté a mi hermano, que me dijo que yo tenía una novia, pero lo dijo por molestar, y empezó como a decir comentarios de que una relación de una mujer y una mujer no se

Quote ID	English Version (as published)	Original Spanish Version
	of started to support him, and I said to her, but why should it be like that if he has to accept anyone else's preferences, whether they like women, men, or if they want to be transgender or whatever, right? It's like men are given this privilege to feel entitled to judge other people's choices, right?. It just feels so, I don't know, sexist… I don't know.	puede y mi mamá como que lo empezó a apoyar y yo le dije, pero por qué si no tendría que ser así si él tiene que aceptar cualquier otro gusto de una persona si le gusta la mujer, el hombre, o sea, si quiere ser transgénero y así, ¿sí? o sea, es como que a los hombres les dan el privilegio y poderse meter por los gustos de otra persona, ¿sí?. Y es como tan, no sé, machista, no sé.
**C4**	Let's remember that before the pandemic, we were coming from a wave of social unrest, not just in Colombia. [...] The pandemic, in a way, was also used to silence those outbreaks of discontent. And now they're reawakening. [...] For example, let's say those on "Primera Línea" (Front Line), and the same with the women from "Ni Una Más" (Not one more), and all those kinds of social movements. They're reawakening and generating, let's say the unrest is reawakening. And with the pandemic, it's let's say, more fuelled, that is , instead of extinguishing it, it generated a glowing ember, which remained there and reappering, but now, without fear. They're, in a way, losing their fear. And that's something that cannot be reversed.	Porque pues recordemos que antes de la pandemia veníamos de un estallido social, no solamente en Colombia […] y pues la pandemia, pues en cierta manera también la utilizaron mucho para apagar esos brotes de inconformidad. Y ahorita están volviendo a despertarse […] por ejemplo, digamos los de los de la primera línea, y lo mismo con las mujeres de ni una más, y todo eso, toda esa serie de este de situaciones sociales. Están volviendo a despertarse y está generando, digamos, se está volviendo a despertar ese malestar. Y con la pandemia, pues está, digamos más atizonado, o sea, en lugar de apagarlo, eso generó como un tizón, que quedó allá y se está volviendo a dar, pero ya como sin miedo, ellos están en cierta manera, perdiendo el miedo. Que es algo que eso no tiene reversa.
